# Dynamic Hydrogen‐Bonding Nanonetworks and Asymmetric Dual‐Interface Built‐In Electric Fields Cooperatively Mediate Proton‐Coupled Electron Transfer for C─H Activation

**DOI:** 10.1002/advs.74462

**Published:** 2026-03-30

**Authors:** Yi‐Wen Han, Run‐Yu Liu, Yu Chen, Lei Ye, Tian‐Jun Gong, Xue‐Bin Lu, Ning Yan, Yao Fu

**Affiliations:** ^1^ State Key Laboratory of Precision and Intelligent Chemistry Anhui Province Key Laboratory of Biomass Chemistry University of Science and Technology of China Hefei China; ^2^ Department of Chemical and Biomolecular Engineering National University of Singapore Singapore Singapore; ^3^ School of Environmental Science and Engineering Tianjin University Tianjin China; ^4^ Centre for Hydrogen Innovations National University of Singapore Singapore Singapore

**Keywords:** biomass valorization via C─H Activation, built‐in electric fields, hollow nanoreactors, photocatalysis, proton–electron dual‐transport‐channel photocatalysts

## Abstract

C─H bond activation represents a ubiquitous transformation in chemistry, yet challenging owing to the complex requirements for proton and electron transfer. A general strategy for constructing proton–electron dual‐transport‐channel photocatalysts: hollow hierarchical Co_3_S_4_/Sv‐chalcogenide/Ti_3_C_2_ nanoreactors (Sv = sulfur vacancies, chalcogenide = CdIn_2_S_4_, ZnIn_2_S_4_, CdS) is developed via lateral epitaxy and defect‐mediated heterocomponent anchorage. These ternary‐component nanoreactors integrate dynamic hydrogen‐bonding nanonetworks and asymmetric dual‐interface built‐in electric fields (BIEFs), acting as the strong proton/electron extractors for steering proton‐coupled electron transfer (PCET) in C─H activation of biomass‐derived molecules. The BIEFs‐induced electron transport channel is featured by powerful photocarrier enrichment and feeble photocarrier recombination at Co_3_S_4_/chalcogenide S‐scheme heterointerface, and photocarrier localization and delocalized‐electron transport at Sv‐chalcogenide/Ti_3_C_2_ Schottky heterointerface. The hydrogen bond network‐induced proton transport channel lies in electron‐enriched interfacial lattice oxygen for mediating the substrate deprotonation via nucleophilic abstraction, and the hydrophilic MXene for guiding proton transfer along modified dynamic hydrogen‐bonding nanonetworks. By virtue of dynamically optimized molecular catalytic behavior accomplished by pivotal intermediate adsorption/activation regulation, representative Co_3_S_4_/Sv‐CdIn_2_S_4_/Ti_3_C_2_ HNR exhibits remarkable C─H activation performance and broad substrate compatibility. This work establishes a pioneering paradigm for manipulating proton–electron dual‐transport‐channel by hydrogen‐bonding nanonetworks and BIEFs, offering novel strategies for regulating molecular catalytic behavior in complex reaction pathways.

## Graphical abstract

The general strategy for constructing proton–electron dual‐transport‐channel photocatalysts (hollow Co_3_S_4_/Sv‐chalcogenide/Ti_3_C_2_ nanoreactors) is developed. Theses ternary‐component nanoreactors feature dynamic hydrogen‐bonding nanonetworks as proton‐transport channels and asymmetric dual‐interface BIEFs as electron‐transport channels, serving as a catalytic platform for activating proton‐coupled electron transfer (PCET) in biomass C─H activation.

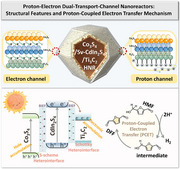

## 1 Introduction

Extensive investigations across diverse systems have revealed that constructing multicomponent heterostructures effectively tailors both the electronic configuration and geometric property of chalcogenides while simultaneously establishing spatially separated oxidation and reduction sites [1, 2, 3]. C─H bond activation constitutes a crucial elementary step in photocatalytic redox transformation (typically, the coproduction of value‐added chemicals and hydrogen) through full utilization of photogenerated electron‐hole pairs, while are pervasive among catalytic chemistry and organic synthesis [4, 5, 6, 7]. Activation of C─H bonds with high bond dissociation energy (BDE), particularly through the decoupled proton transfer (PT) and electron transfer (ET) processes in stepwise electron–proton transfer mechanisms, leads to sequential energy barriers (ΔG_PT_ + ΔG_ET_) [8] that pose significant kinetic challenges. Proton‐coupled electron transfer (PCET) [9], by circumventing the formation of high‐energy intermediates [9, 10, 11], confers thermodynamic and kinetic advantages to a myriad of challenging catalytic transformation [12, 13], including CO_2_RR [14], ORR [15], NRR [16]. The semiconductor photocatalysis for C─H bond activation gained substantial advancements with inherent limitations in fundamental mechanistic elucidation, exemplified by PCET pathways [6]. Therefore, the rational design and precise fabrication of advanced heterostructure photocatalysts, coupled with the establishment of a well‐defined structure‐PCET property‐performacne relationship, are essential for advancing C─H bond activation.

PCET process benefits from the synergistic construction of physical electron transport channels and proton transport channels within the catalyst architecture. Concerning electron transport management, the built‐in electric field (BIEFs), architected via asymmetric charge redistribution, induces localized charge polarization through electric field‐directed segregation of negative‐positive charges and the counter‐directional interfacial transport, thereby establishing a continuous potential gradient for charge transport promotion [17, 18, 19, 20, 21]. The effectiveness of governing electron transport channels through strategic BIEF regulation (e.g., modulating intrinsic semiconductor lattice distortions [22, 23, 24, 25] and engineering hetero‐component interfacial interactions [26]) has been validated in various architectures, including tandem BIEFs, full‐space BIEFs, and dual‐interface BIEFs [2, 27, 28]. Concerning proton transport management, materials featuring hydrophilic ligands (e.g., ─OH/─O), such as MXene, demonstrate remarkable proton conduction potential in aqueous systems by leveraging their exceptional hydrogen‐bond‐forming ability [29, 30], which acts synergistically with the dynamic hydrogen‐bonding nanonetworks in the Grotthuss mechanism [15]. Additionally, structurally engineered bridging heteroatoms (X = O, N, S) at atomic interfaces serve as electron‐enriched centers, which not only promote crucial deprotonation through nucleophilic activation but also stabilize protonated intermediates by selective adsorption [31]. Proton transport, therefore, fundamentally depends on such positioned functional sites and communicating hydrogen‐bond networks. Ternary heterostructures, characterized by spatially specific ternary‐component systems and asymmetric surface chemistry, provide an ideal platform for constructing multiple proton–electron transport channels, leveraging their multi‐component tunability and multi‐interface synergistic properties. Building upon the considerations, we foresee that constructing a ternary‐component ordered‐space integration architecture (featuring Co_3_S_4_ core‐chalcogenide shell‐MXene immobilization) as a molecular platform, atomic‐scale dual‐interface control (for electron transfer) and dynamic hydrogen‐bonding nanonetworks (for proton transfer) can be regulated on it to enable electron‐proton dual‐transport‐channel establishment. Moreover, utilizing spatiotemporal‐scale characterization techniques elucidate the PCET mechanism underlying C─H bond activation.

Herein, the hollow hierarchical Co_3_S_4_/Sv‐chalcogenide/Ti_3_C_2_ nanoreactors with spatially segregated proton–electron dual‐transport channels are developed, which act as catalytic platforms to support PCET in photocatalytic C─H activation. The representative Co_3_S_4_/Sv‐CdIn_2_S_4_/Ti_3_C_2_ hollow nanoreactor (HNR) features dynamic hydrogen‐bonding nanonetworks as proton‐transport channels and asymmetric dual‐interface BIEFs as electron‐transport channels, as validated by X‐ray absorption spectroscopy (XAS), Kelvin probe force microscopy (KPFM), two‐electrode AC impedance spectroscopy technique. Within this heterostructured architecture, electron transport proceeds through the core framework concomitant with enhanced proton transfer mediated by terminal functional groups, collectively enabling dynamically matched molecular catalytic behavior and culminating in exceptional performance metrics (659 µmol·g^−1^·h^−1^ activity, 91.3% selectivity, DFF) for C─H activation of biomass‐derived molecules (nine examples). A combination of in situ diffuse reflectance infrared fourier transform spectroscopy (in situ DRIFTS), in situ electron paramagnetic resonance (in situ EPR), in situ X‐ray photoelectron spectroscopy (in situ XPS), transient absorption spectroscopy (fs‐TAS), and density functional theory (DFT) simulation provides multidimensional visualization of PCET process.

## 2 Results and Discussion

### 2.1 Establishing Proton–Electron Dual‐Transport‐Channel Photocatalysts with Asymmetric Dual‐Interface BIEFs and Proton‐Conductive Surface Structure

This strategy constructs ternary‐component heterostructures while simultaneously establishing proton–electron dual‐transport channels for the following reasons: (**1) Lateral epitaxy for hollow core–shell Co_3_S_4_/chalcogenide S‐scheme heterostructure fabrication**. The amorphous cobalt sulfide (denoted as CoS_x_) core layer proceeds via thioacetamide (TAA)‐mediated ZIF‐67 dodecahedra etching, where sulfide ions liberated from TAA diffusion undergo dual‐phase infiltration, simultaneously permeating external matrices and penetrating internal architectures to coordinate with metal species during framework decomposition. Subsequent lateral epitaxial growth of chalcogenides (such as CdIn_2_S_4_) on the CoS_x_ outermost surface proceeds through dual nucleation pathways, wherein S^2−^ anions and Cd^2+^/In^3+^ cations undergo both heterogeneous nucleation on CoS_x_ substrates and spontaneous homogeneous nucleation. These two particles, Co_3_S_4_/CdIn_2_S_4_ nanocomposites and CdIn_2_S_4_ nanoparticles, then self‐assemble to architecturally form a large ortho‐dodecahedral hollow core–shell nanocage. **(2) Defect‐mediated heterocomponent anchorage for Sv‐chalcogenide/Ti_3_C_2_ Schottky heterojunction construction**. The Ti_3_C_2_ MXene are immobilized at core–shell architectures (chalcogenide side) through interface M─O (Metal–Oxygen) chemical bonds. The defect‐rich domains within pre‐modulated CdIn_2_S_4_, characterized by abundant coordinative unsaturation atoms and delocalized local electrons, function as strategic anchoring centers for establishing robust interfacial bonding with Ti_3_C_2_ MXene. The ternary heterostructure, which integrates an S‐scheme heterojunction at the Co_3_S_4_/CdIn_2_S_4_ interface and a Schottky junction at the Sv‐CdIn_2_S_4_/Ti_3_C_2_ interface, establishes an asymmetric bidirectional BIEFs with CdIn_2_S_4_ as the central mediator, creating potential electron transport channels. The dynamic hydrogen‐bonding network formed between hydrophilic terminal groups (e.g., ─OH) on MXene surfaces and water molecules establishes a transient yet continuously reconfigurable bonding system, enabling dynamically balanced proton conduction pathways through reversible hydrogen bond rupture and reformation.

**FIGURE 1:**
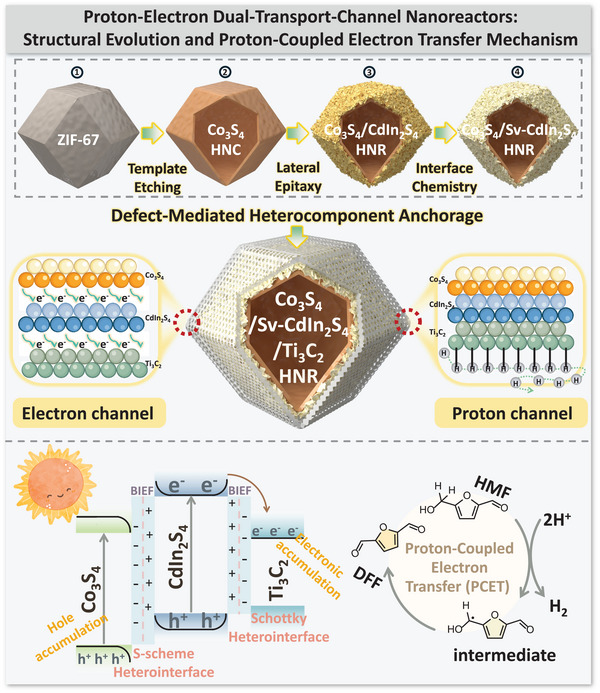
Schematic representation illustrating the synthetic procedures and distinctive structures of proton–electron dual‐transport‐channel nanoreactors (Co_3_S_4_/Sv‐chalcogenide/Ti_3_C_2_), and the nanoreactor‐driven PCET in photocatalytic C─H activation.

The structural evolution of representative Co_3_S_4_/Sv‐CdIn_2_S_4_/Ti_3_C_2_ HNR is depicted in Figure 1. Detailed synthetic information about **Co_3_S_4_ HNC** (the hollow Co_3_S_4_ nanocage), **Co_3_S_4_/CdIn_2_S_4_ HNR** (the hollow core–shell Co_3_S_4_/CdIn_2_S_4_ nanoreactor), **Co_3_S_4_/Sv‐CdIn_2_S_4_ HNR** (the Co_3_S_4_/CdIn_2_S_4_ HNR with sulfur vacancies in CdIn_2_S_4_), **Co_3_S_4_/Sv‐CdIn_2_S_4_/Ti_3_C_2_ HNR** (the hollow Co_3_S_4_/CdIn_2_S_4_/Ti_3_C_2_ nanoreactor with sulfur vacancies in CdIn_2_S_4_ and interfacial M‐O bonds between Sv‐CdIn_2_S_4_ and Ti_3_C_2_), and **Co_3_S_4_/Sv‐chalcogenide/Ti_3_C_2_** can be accessed in Supporting Information.

### 2.2 Distinctive Nanostructures, Chemical Compositions, and Optical Absorption Properties

The distinctive micro‐architectures (e.g., hollow core–shell‐surface immobilization ternary‐component heterostructures, MXene surface functionalization, lattice defects, interfacial chemical bonds), chemical compositions, and optical absorption properties of the model Co_3_S_4_/Sv‐CdIn_2_S_4_/Ti_3_C_2_ HNR were investigated as fundamental for studying intrinsic structure‐property correlations.

**FIGURE 2:**
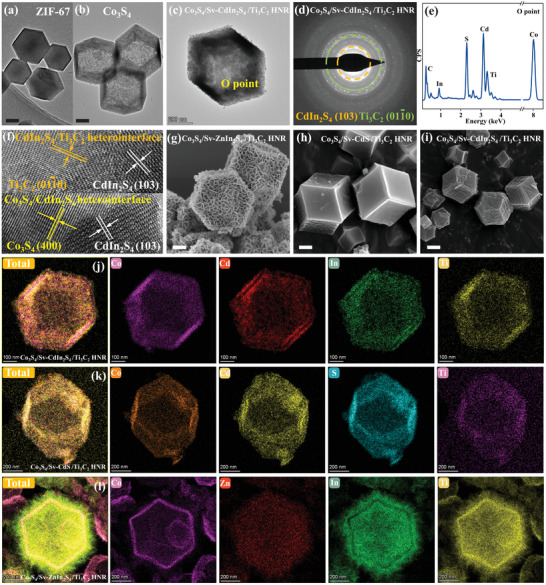
TEM image of a) ZIF‐67, b) Co_3_S_4_, c) Co_3_S_4_/Sv‐CdIn_2_S_4_/Ti_3_C_2_ HNR. SAED image of d) Co_3_S_4_/Sv‐ZnIn_2_S_4_/Ti_3_C_2_ HNR. e) EDX spectrum measured at the location marked ‘O’ in (c). f) HRTEM image of Co_3_S_4_/Sv‐CdIn_2_S_4_/Ti_3_C_2_ HNR. TEM image of g) Co_3_S_4_/Sv‐ZnIn_2_S_4_/Ti_3_C_2_ HNR; h) Co_3_S_4_/Sv‐CdS/Ti_3_C_2_ HNR; i) Co_3_S_4_/Sv‐CdIn_2_S_4_/Ti_3_C_2_ HNR. Corresponding HAADF‐STEM image and elemental mapping of them (j–l). Scale bars: a) 100 nm, b) 100 nm, g) 100 nm, h) 100 nm, i) 200 nm.

The ternary core–shell‐surface immobilization heterostructure evolution was identified by morphological characterization and its derivative analyses. Transmission electron microscopy (TEM) shows that the synthetic ZIF‐67 precursor undergoes a TAA‐mediated sulfidation and etching process, evolving into a hollow Co_3_S_4_ structure (Figure 2a,b). The 3D core–shell nanocage (Co_3_S_4_/CdIn_2_S_4_ HNR) maintains a dodecahedral hollow architecture through the lateral epitaxy of CdIn_2_S_4_ on the Co_3_S_4_ core surface, driven by the assembly of heterogeneous Co_3_S_4_/CdIn_2_S_4_ nanocomposites and homogeneous CdIn_2_S_4_ nanoparticles. The defect‐anchored Ti_3_C_2_ MXene uniformly dispersed on the CdIn_2_S_4_ surface enables the preservation of the ternary‐component heterostructure's hollow core–shell morphology (Figure 2c). Compositional details of the selected area (O in Figure 2c) within the Co_3_S_4_/Sv‐CdIn_2_S_4_/Ti_3_C_2_ HNR were determined via energy‐dispersive X‐ray spectroscopy (EDX) analysis (Figure 2e). The combination of selected area electron diffraction (SAED) and high‐resolution TEM (HRTEM) allowed for the clear identification of the (400) [32], (103) [33], (0110) [34] crystallographic planes from Co_3_S_4_, CdIn_2_S_4_, Ti_3_C_2_, respectively, within the Co_3_S_4_/Sv‐CdIn_2_S_4_/Ti_3_C_2_ (Figure 2d,f). The broad applicability of this versatile assembly strategy to multiple chalcogenide‐based ternary‐component heterostructures (e.g., CdIn_2_S_4_, CdS, ZnIn_2_S_4_) is evidenced by their corresponding TEM morphology and high‐angle annular dark field‐scanning transmission electron microscopy (HAADF‐STEM) elemental distribution profiles, shown in Figure 2g–l.

**FIGURE 3:**
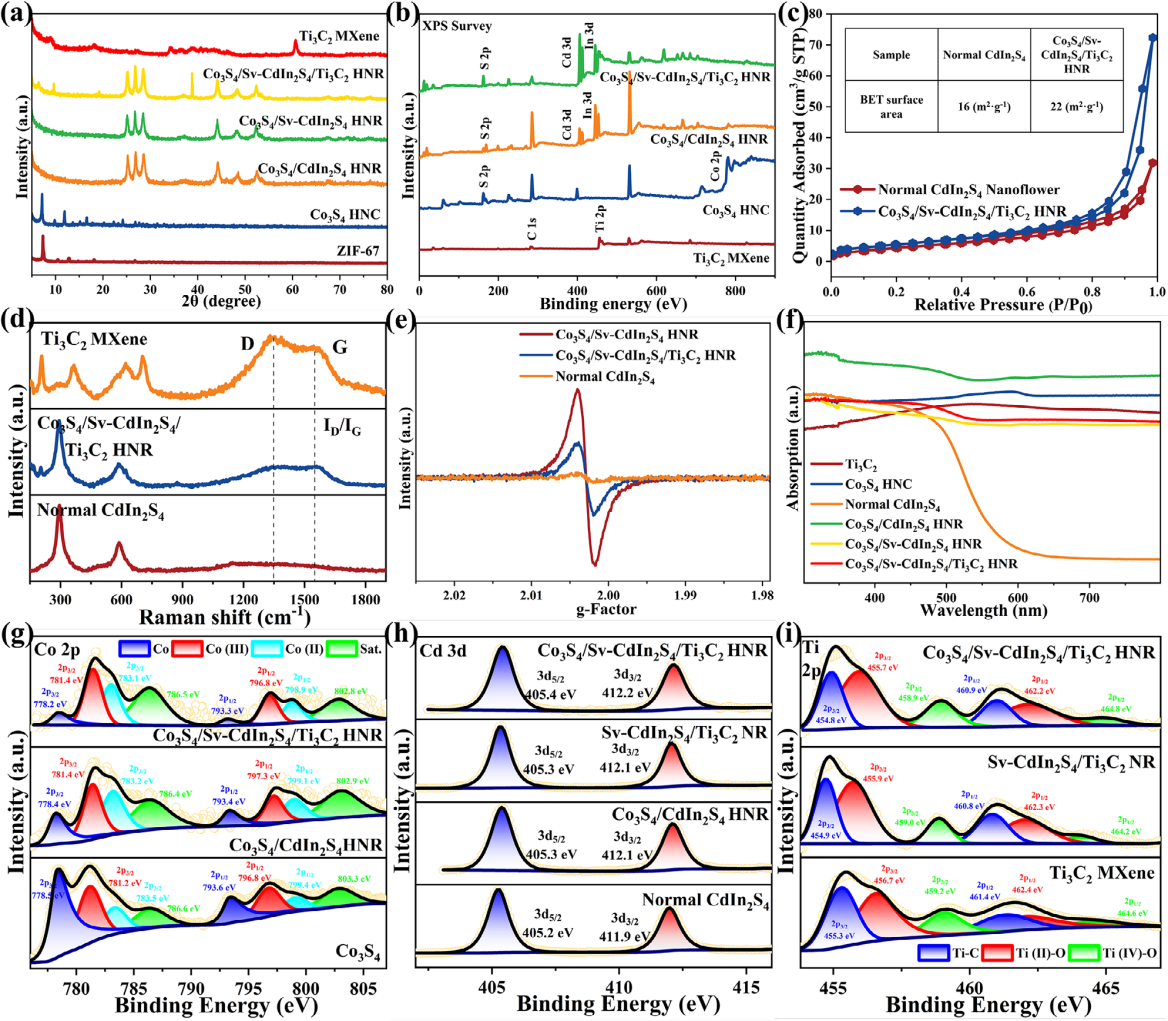
a) XRD patterns, b) XPS survey, c) BET analysis, d) Raman spectra, e) EPR spectra, f) UV/Vis DRS spectra of as‐synthesized materials. High‐resolution XPS spectra of g) Co 2p, h) Cd 3d, and i) Ti 2p of as‐synthesized materials.

**FIGURE 4:**
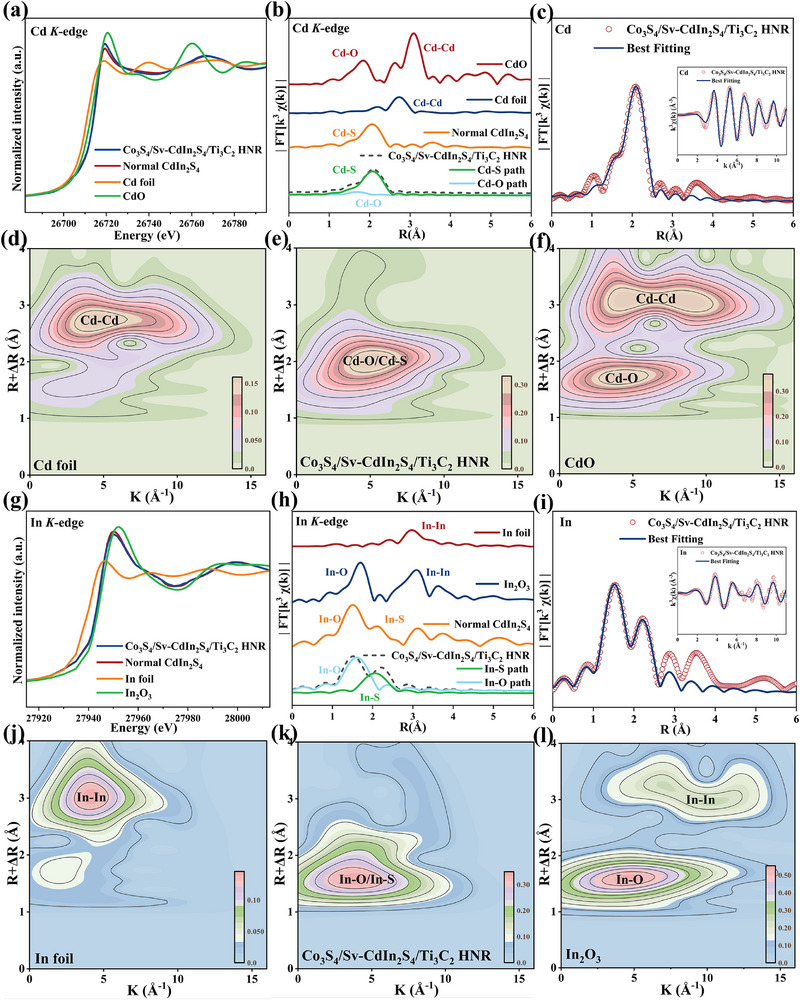
Normalized a) Cd and g) In K‐edge XANES. The *k* ^3^‐weighted FT‐EXAFS spectra of Normal CdIn_2_S_4_ and Co_3_S_4_/Sv‐CdIn_2_S_4_/Ti_3_C_2_ HNR in b) Cd K‐edge and h) In K‐edge. EXAFS spectra of c) Cd K‐edge and i) In K‐edge of Co_3_S_4_/Sv‐CdIn_2_S_4_/Ti_3_C_2_ HNR in R space and k space (inside). Wavelet transforms of d–f) Cd foil, Co_3_S_4_/Sv‐CdIn_2_S_4_/Ti_3_C_2_ HNR, CdO and j–l) In foil, Co_3_S_4_/Sv‐CdIn_2_S_4_/Ti_3_C_2_ HNR, In_2_O_3_.

Surface/interfacial chemistry and lattice defects were identified. XPS analysis (Figure 3i) identifies distinct Ti 2p signals (six deconvoluted peaks) corresponding to Ti^2+^─O (455.7 eV, 2p_3/2_ and 462.2 eV, 2p_1/2_) and Ti^4+^─O (458.9 eV, 2p_3/2_ and 464.8 eV, 2p_1/2_) oxidation states, and Ti─C species. These findings, coupled with Fourier transform infrared spectroscopy (FTIR) spectroscopy (Figure S1) that displays characteristic hydroxyl (─OH) stretching vibrations, provide conclusive evidence for MXene surface functionalization (─OH). XAS provides clear evidence for the establishment of interfacial M─O chemical bonds between core–shell architectures and surface‐functionalized MXene (Figure 4a–l; Figures S2 and S3, Tables S1 and S2). X‐ray absorption near‐edge structure (XANES) spectra exhibit a pre‐edge peak centered at 26711/27938 eV, consistent with orbital hybridization between Cd^2+^/In^3+^ and oxygen atoms. Fourier‐transformed (FT) *k* ^3^‐weighted extended X‐ray absorption fine structure (EXAFS) profiles in R‐space reveal prominent peaks at 1.82 and 1.78 Å in the Cd and In K‐edges of the Co_3_S_4_/Sv‐CdIn_2_S_4_/Ti_3_C_2_ HNR, respectively, consistent with first‐shell Cd─O and In─O coordination in the first shell. These bond distances are analogous to those in CdO and In_2_O_3_, yet notably shorter than the Cd─Cd/In─In metallic and Cd─S/In─S sulfide paths observed in corresponding metal foil and pristine Normal CdIn_2_S_4_. references. FT‐EXAFS and wavelet transform (WT) indicate a reduction in Cd─S and In─S signal intensities resulting from decreased coordination numbers of Zn and In, alongside an enhancement in Cd─O and In─O intensities attributable to oxygen incorporation at defect sites (a pronounced Cd─O─Ti/In─O─Ti three‐body correlation signal), with first‐shell EXAFS fitting performed based on Cd─O and In─O scattering paths in both R and k spaces. In EPR spectroscopy (Figure 3e), the quantified lattice defect concentration is proportional to the characteristic peak intensity. The engineered lattice defects in Co_3_S_4_/CdIn_2_S_4_ HNRs yield significantly intensified characteristic EPR signals (g = 2.004) compared to pristine CdIn_2_S_4_. Subsequent Ti_3_C_2_ MXene immobilization on the core–shell surface partially passivates these defects through chemical bonding between terminal oxygen atoms of Ti_3_C_2_ MXene and coordinatively unsaturated metal sites in CdIn_2_S_4_ shell.

Finally, the crystal structures and optical properties were elucidated. X‐ray diffraction (XRD) analysis (Figure 3a) shows the crystalline phase evolution during ternary‐component heterostructure (Co_3_S_4_/Sv‐CdIn_2_S_4_/Ti_3_C_2_ HNR) establishment, with diffraction patterns matching reference standards of Co_3_S_4_ (JCPDS 42–1448) and CdIn_2_S_4_ (JCPDS 27–0060). Brunauer–Emmett–Teller (BET) measurements demonstrate that the hollow architecture contributes to the enhanced specific surface area of the material (Figure 3c). Raman spectroscopy confirms the robust bonding between Ti_3_C_2_‐terminal oxygen and unsaturated metal atom in Sv‐CdIn_2_S_4_ (evidenced by altered I_D_/I_G_ ratio, Figure 3d). UV‐vis diffuse reflectance spectroscopy (UV–vis DRS) evidenced that the integration of Ti_3_C_2_ and Co_3_S_4_, as excellent photosensitive components, enabled the CdIn_2_S_4_‐based composite to achieve full‐spectrum photoresponse from the UV to visible light regions (Figure 3f). Tauc plot analysis (Figures S4 and S5) yields energy bandgap (Eg) of 2.2 eV for Normal CdIn_2_S_4_ and 1.53 eV for Co_3_S_4_, corresponding Mott‐Schottky measurements reveal flat band potentials of −0.64 and −0.97 V (vs. NHE) for the two materials, respectively. Consistent with typical n‐type semiconductor behavior, the conduction band potentials (E_CB_) are situated near these flat band potentials [35].

Multiscale structural characterization confirms that the Co_3_S_4_/Sv‐CdIn_2_S_4_/Ti_3_C_2_ heterostructure, featuring a meticulously engineered dual‐interface design, possesses a well‐defined ternary microstructure (Co_3_S_4_ core – CdIn_2_S_4_ shell – surface immobilization Ti_3_C_2_). These architectures endow the material with the potential to serve as a proton/electron extractor and relay station in catalytic applications.

### 2.3 Superior Performances of Ternary‐Component Heterostructures for Photocatalytic C─H Activation

**FIGURE 5:**
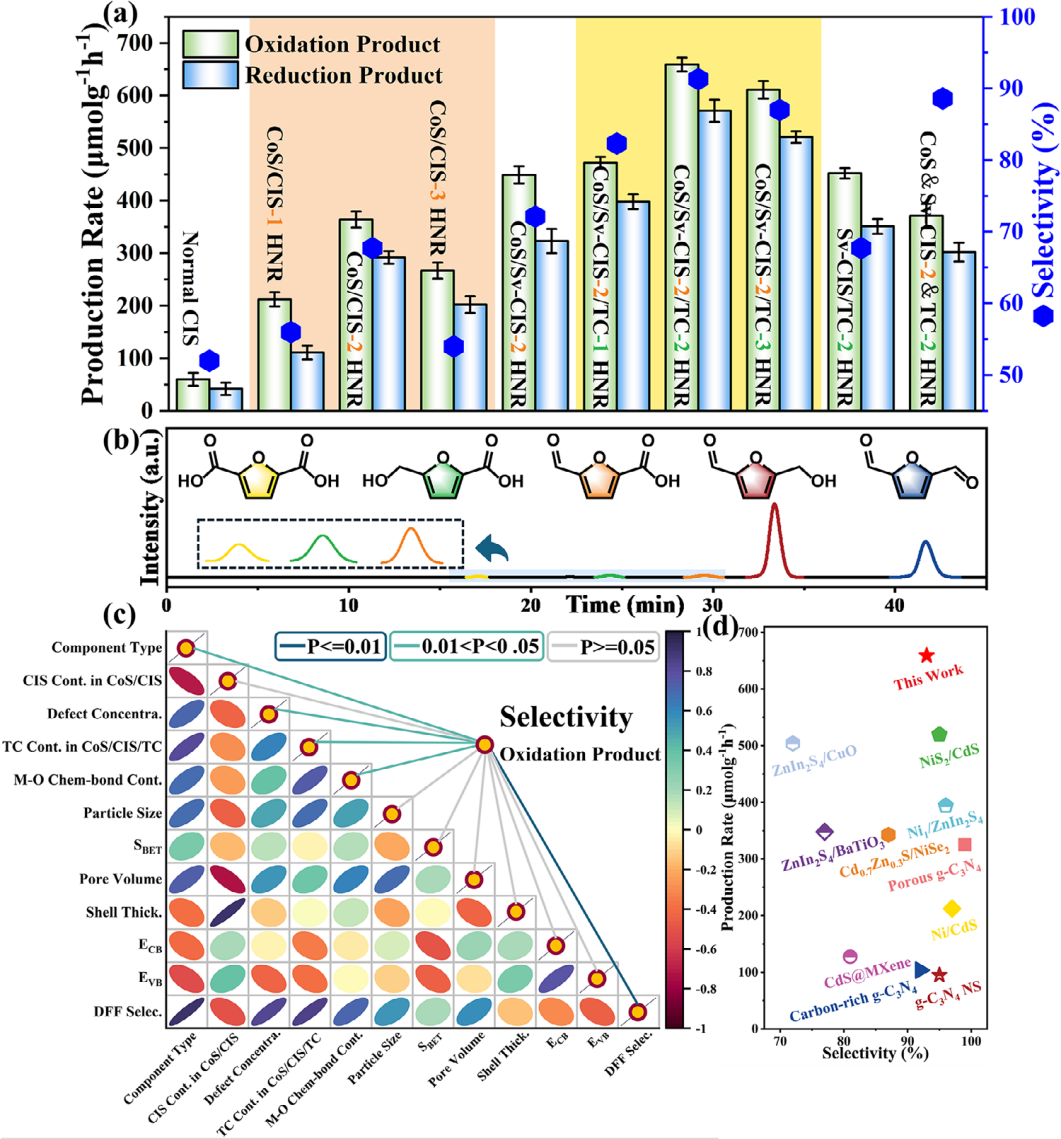
a) Visible‐light‐irradiation C─H bond activation performance on various photocatalysts. b) HPLC analysis of the reaction mixture. c) Heat map analysis of the physicochemical property‐selectivity relationship in photocatalysis. d) Comparison of Co_3_S_4_/Sv‐CdIn_2_S_4_‐2/Ti_3_C_2_‐2 HNR's performance (activity and selectivity) in the manuscript and reported HMF‐to‐DFF conversion systems.

Visible‐light‐irradiation C─H bond activation performance was examined using 5‐ hydroxymethylfurfural (HMF), a representative biomass platform molecule, as a model compound over a series of as‐prepared nanoreactors with proton transport channels, electron transport channels, proton–electron dual transport channels, with liquid and gaseous products detection by high‐performance liquid chromatography (HPLC) and gas chromatography (GC). The 2,5‐diformylfuran (DFF) is identified as the main product, with trace amounts of 5‐hydroxymethyl‐2‐furancarboxylic acid (HMFCA), 5‐formyl‐2‐furancarboxylic acid (FFCA), and 2,5‐furandicarboxylic acid (FDCA) generated as byproducts among the liquid products (Figure 5b). The enhancement in catalytic performance exhibits a positive correlation with several structural factors: the hollow core–shell nanoarchitecture, the hetero‐components type (ranging from mono‐ to tri‐component), the interfacial electron transport channel configuration, and the surface proton transport channel configuration. Following optimization of binary (Co_3_S_4_ and CdIn_2_S_4_) and ternary (Co_3_S_4_/Sv‐CdIn_2_S_4_ and Ti_3_C_2_) structural ratios, the representative Co_3_S_4_/Sv‐CdIn_2_S_4_‐2/Ti_3_C_2_‐2 HNR heterostructure exhibits exceptional cooperative photoredox catalytic activity, with DFF and H_2_ production rates reaching 659 and 571 µmol·g^−1^·h^−1^, respectively, surpassing those of pristine CdIn_2_S_4_ and its physical mixture counterpart (with identical composition) by factors of 13.5 and 1.78 (Figure 5a; Figure S6). Building upon the universally high product selectivity observed across all proton–electron dual‐transport‐channel catalysts, a systematic investigation was conducted into the structure‐productivity relationship between DFF selectivity and the physicochemical properties of the catalysts. Pearson correlation analysis based on advanced spectroscopic characterization shows that the selectivity exhibits a positive correlation with the component type, lattice defect concentration, and interfacial M─O chemical bonds content (Figure 5c). Encouraged by the satisfactory productivity and selectivity (Figure 5d), recycling experiments were conducted, demonstrating maintained catalytic activity over five consecutive cycles (Figure S7).

### 2.4 Electron Flow Directed by Asymmetric Dual‐Interface BIEFs and Proton Relay Enabled by Hydrogen‐Bonding Nanonetworks

**FIGURE 6:**
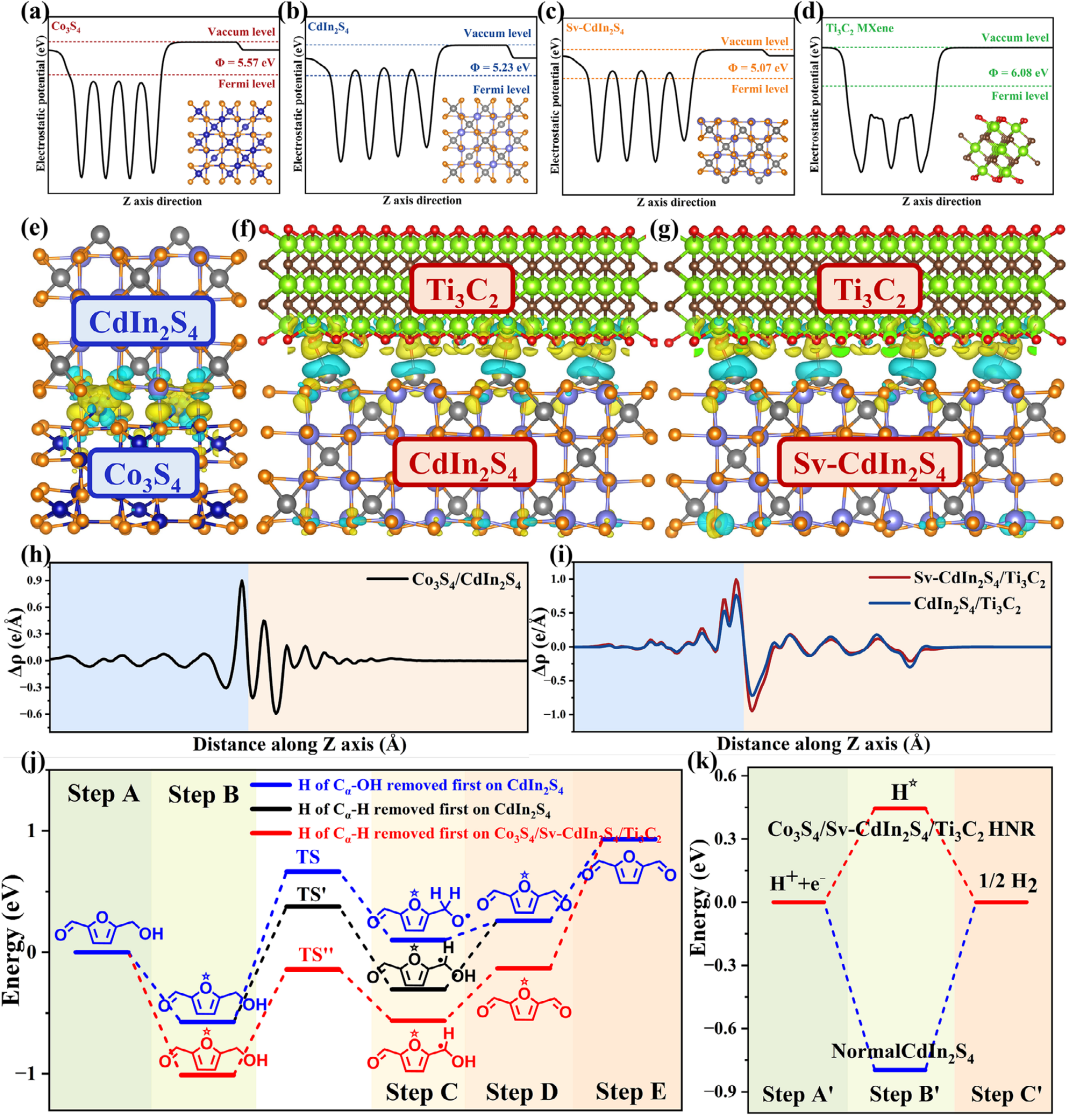
Work function and electrostatic potential of a) Co_3_S_4_, b) CdIn_2_S_4_, c) Sv‐CdIn_2_S_4,_ and d) Ti_3_C_2_ MXene. The calculated electron density differences of e) Co_3_S_4_/Sv‐CdIn_2_S_4_ and f) CdIn_2_S_4_/Ti_3_C_2_ and g) Sv‐CdIn_2_S_4_/Ti_3_C_2_. Planar‐averaged electron density differences along Z direction of h) Co_3_S_4_/CdIn_2_S_4_ and i) CdIn_2_S_4_/Ti_3_C_2_ & Sv‐CdIn_2_S_4_/Ti_3_C_2._ The simulated energy diagram of photocatalytic j) HMF‐to‐DFF and k) hydrogen evolution over Co_3_S_4_/Sv‐CdIn_2_S_4_/Ti_3_C_2_ HNR and Normal CdIn_2_S_4_.

To investigate artificially modulated electron transfer channels in ternary heterostructures, the dual BIEFs construction at the Co_3_S_4_/Sv‐CdIn_2_S_4_/Ti_3_C_2_ HNR interface (Co_3_S_4_/CdIn_2_S_4_ and Sv‐CdIn_2_S_4_/Ti_3_C_2_ interfacial BIEFs), the BIEF‐induced charge transfer pathways and BIEF‐boosted charge transfer kinetics, were systematically investigated through multiscale characterization and theoretical simulations (Figure 6).

**FIGURE 7:**
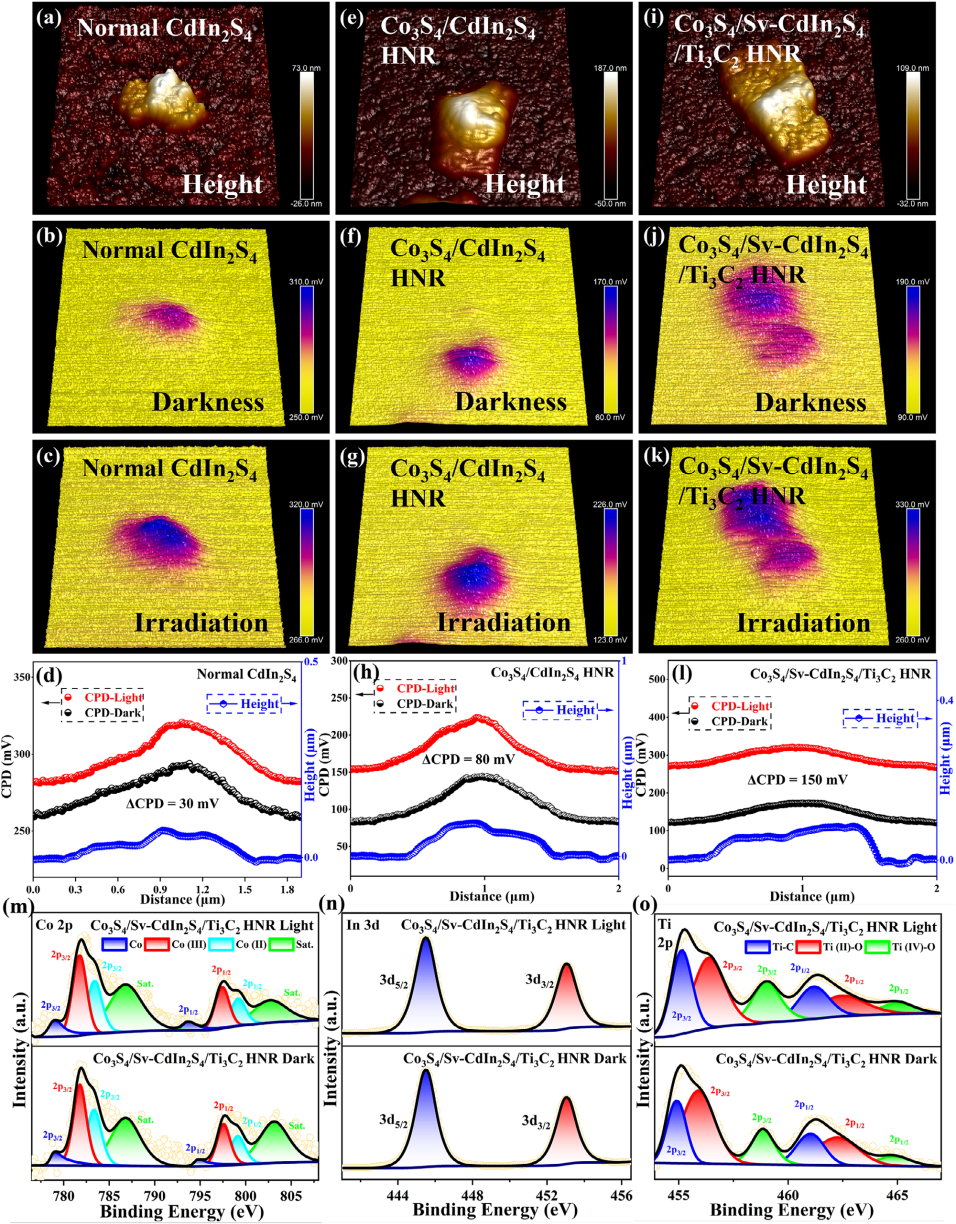
AFM images, 3D surface potential distribution images, photoinduced ΔCPD along with the height profiles over a–d) Normal CdIn_2_S_4_, e–h) Co_3_S_4_/CdIn_2_S_4_ HNR, and i–l) Co_3_S_4_/Sv‐CdIn_2_S_4_/Ti_3_C_2_ HNR. Darkness/irradiation XPS spectra for m) Co 2p, n) In 3d, o) Ti 2p over Co_3_S_4_/Sv‐CdIn_2_S_4_/Ti_3_C_2_ HNR.

**FIGURE 8:**
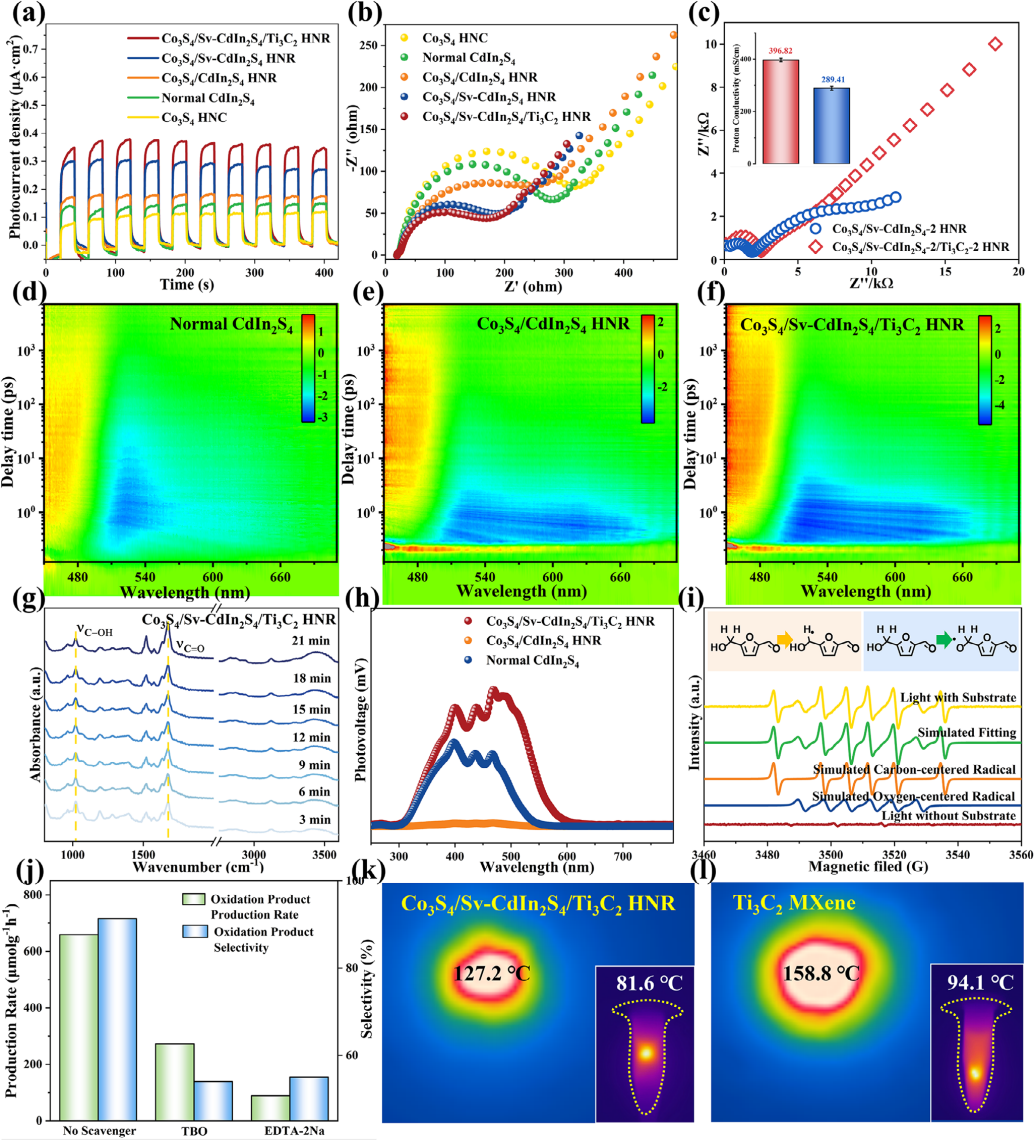
a) TPC response spectra and b) EIS Nyquist plots c) two‐electrode AC impedance spectroscopy measurement of as‐prepared photocatalysts. The 2D transient absorption surface plots of d) Normal CdIn_2_S_4_, e) Co_3_S_4_/CdIn_2_S_4_ HNR, f) Co_3_S_4_/Sv‐CdIn_2_S_4_/Ti_3_C_2_ HNR. g) In situ DRIFTS spectra of HMF adsorption over Co_3_S_4_/Sv‐CdIn_2_S_4_/Ti_3_C_2_ HNR. h) SPV spectra of synthetic samples. i) In situ EPR spectra for photocatalytic redox mechanism investigation. j) Capture agent experiments. Surface temperature measurement of k) Co_3_S_4_/Sv‐CdIn_2_S_4_/Ti_3_C_2_ HNR, and l) Ti_3_C_2_ MXene.

KPFM quantitatively resolves the BIEF strength in the dual interface of Co_3_S_4_/Sv‐CdIn_2_S_4_/Ti_3_C_2_ HNR, a finding corroborated by the correlated surface charge distribution and corresponding height profiles (Figure 7a–l). Dark‐state 3D surface potential mapping reveals inherent asymmetrical spatial charge distribution across Normal CdIn_2_S_4_, Co_3_S_4_/CdIn_2_S_4_ HNR, and Co_3_S_4_/Sv‐CdIn_2_S_4_/Ti_3_C_2_ HNR. Upon illumination, the Co_3_S_4_/CdIn_2_S_4_ HNR demonstrates a markedly higher contact potential difference variation (ΔCPD = 80 mV) than that of Normal CdIn_2_S_4_ (30 mV), and further enhancement is achieved in the Co_3_S_4_/Sv‐CdIn_2_S_4_/Ti_3_C_2_ HNR, which exhibits a maximal ΔCPD of 150 mV. Surface photovoltage (SPV) spectroscopy directly probes band bending via interfacial charge transfer in photocatalysts. The SPV intensity scales monotonically with surface electron‐transfer efficiency, yielding the activity trend: Co_3_S_4_/Sv‐CdIn_2_S_4_/Ti_3_C_2_ HNR > Co_3_S_4_/CdIn_2_S_4_ HNR > Normal CdIn_2_S_4_ (Figure 8h). DFT electronic structure simulations (including density of states (DOS), work functions) reveal that the interfacial BIEF originates from the work function (Φ) gradient discrepancy at the Co_3_S_4_/CdIn_2_S_4_ and Sv‐CdIn_2_S_4_/Ti_3_C_2_ interface (Figure 6a–d; Figure S8), which provides an intrinsic driving force for directional charge migration.

Asymmetric dual‐interface BIEFs, constructed through dual‐side atomic‐level interface modulation, induces unique charge transfer pathways. High‐resolution X‐ray photoelectron spectroscopy (HRXPS) analysis reveals distinct core‐level shifts in as‐prepared heterostructures: both Co_3_S_4_/CdIn_2_S_4_ and Sv‐CdIn_2_S_4_/Ti_3_C_2_ exhibit positive binding energy shifts in Cd 3d spectra relative to pristine Normal CdIn_2_S_4_, while Co 2p in Co_3_S_4_/CdIn_2_S_4_ and Ti 2p in Sv‐CdIn_2_S_4_/Ti_3_C_2_ show negative shifts compared to Co_3_S_4_ and Ti_3_C_2_, respectively. The ternary Co_3_S_4_/Sv‐CdIn_2_S_4_/Ti_3_C_2_ HNR demonstrates further enhanced shifts, additional Cd 3d positive shift, and Ti 2p negative shift, providing direct evidence of strengthened interfacial electronic coupling among all three components (Co_3_S_4_, Sv‐CdIn_2_S_4_, Ti_3_C_2_) at the atomic level (Figure 3g–i). Darkness/irradiation XPS measurements reveal charge redistribution in ternary Co_3_S_4_/Sv‐CdIn_2_S_4_/Ti_3_C_2_ photocatalyst: the negative In 3d and positive Co 2p binding energy shifts confirm photogenerated electron transfer from Co_3_S_4_ to CdIn_2_S_4_, while the negative Ti 2p shift evidences electron migration from CdIn_2_S_4_ to Ti_3_C_2_ (Figure 7m–o). Drawing from the preceding analyses, a ternary Co_3_S_4_/Sv‐CdIn_2_S_4_/Ti_3_C_2_ HNR heterostructure integrates a Co_3_S_4_/CdIn_2_S_4_ S‐scheme heterojunction and a Sv‐CdIn_2_S_4_/Ti_3_C_2_ Schottky junction, At the Co_3_S_4_/CdIn_2_S_4_ interface, Fermi‐level equilibration drives electron transfer from CdIn_2_S_4_ to Co_3_S_4_, generating a CdIn_2_S_4_→Co_3_S_4_ BIEFs. This induces downward band bending in Co_3_S_4_ (due to electron accumulation) and upward band bending in CdIn_2_S_4_ (due to electron depletion). Under photoexcitation, feeble photocarrier recombination between Co_3_S_4_ CB and CdIn_2_S_4_ VB at the heterointerface, thereby preserving powerful photocarrier enrichment in Co_3_S_4_ VB and CdIn_2_S_4_ CB. At the Sv‐CdIn_2_S_4_/Ti_3_C_2_ interface, electron migration from CdIn_2_S_4_ to Ti_3_C_2_ at the Schottky heterojunction interface establishes a CdIn_2_S_4_→Ti_3_C_2_ BIEF, concurrently inducing upward band bending in CdIn_2_S_4_ to form a Schottky barrier. They share CdIn_2_S_4_ as a central mediator, establishing asymmetric dual‐interface BIEFs that direct photogenerated holes toward Co_3_S_4_ and electrons toward Ti_3_C_2_.

This BIEFs accelerates charge transfer kinetics across the heterointerfaces. Photoelectrochemical analyses (steady‐state photoluminescence (PL) peak intensity, time‐resolved PL charge lifetime, transient photocurrent (TPC) density, electrochemistry impedance spectroscopy (EIS) arc radius) collectively demonstrate that dual‐interface BIEFs induce asymmetric charge distribution in Co_3_S_4_/Sv‐CdIn_2_S_4_/Ti_3_C_2_ HNR (Figure 8a,b; Figures S9 and S10), establishing interfacial charge transport channels and enhancing migration efficiency. The fs‐TAS with an advanced pump‐probe setup directly resolves modulated photocarrier dynamics (Figure 8d–f; Figure S11) [36]. The ground‐state bleaching (GSB) signal quantifies photoexcited electron density [36], while excited‐state absorption (ESA) originates from light absorption by excited‐state electrons [37]. The TA kinetic decay profiles confirm accelerated interfacial electron transfer in Co_3_S_4_/Sv‐CdIn_2_S_4_/Ti_3_C_2_ HNR heterostructure [38]. Photothermal energy conversion is provided in the Supporting Information (Figure 8k,l; Figure S12).

To probe artificially modulated proton transfer channels in ternary heterostructures, proton conductivity of as‐prepared materials is measured and calculated using a two‐electrode AC impedance spectroscopy technique. Proton transport measurement reveals that terminal groups proton transfer follows the Newtonian pendulum model at the atomic level [39]. Electrochemical impedance analysis based on Nyquist plots (Figure 8c) demonstrates a proton conductivity trend: Co_3_S_4_/Sv‐CdIn_2_S_4_/Ti_3_C_2_ HNR > Co_3_S_4_/Sv‐CdIn_2_S_4_ HNR. The Co_3_S_4_/Sv‐CdIn_2_S_4_/Ti_3_C_2_ HNR exhibits a proton conductivity of 396.82 mS·cm^−1^ at 30°C, significantly higher than that of Normal CdIn_2_S_4_ (289.41 mS·cm^−^^1^), with the contribution from the MXene's hydrophilic terminal groups to proton conductivity is ≈37%.

The experimental findings visually demonstrate the existence of proton–electron dual‐transport channels within Co_3_S_4_/Sv‐chalcogenide/Ti_3_C_2_ HNR.

### 2.5 Proton–Electron Dual‐Transfer‐Channel Nanoreactors‐Induced Photocatalytic PCET Mechanism

The PCET mechanism induced by proton–electron dual‐transfer‐channel nanoreactors (Co_3_S_4_/Sv‐CdIn_2_S_4_/Ti_3_C_2_ HNR) in C─H bond activation was systematically investigated through scavenger experiments, in situ EPR, in situ DRIFTS, and theoretical simulations.

Initial characterization of the C─H activition system identified key reactive oxygen species (ROS) and radical intermediates. Scavenging experiments (Figure 8j) using tert‐butanol (TBO) for hydroxyl radicals (•OH) quenching and EDTA‐2Na for hole (h^+^) trapping were implemented, with unscavenged control systems monitored simultaneously for mechanistic benchmarking. EDTA‐2Na exhibits superior inhibitory efficacy over TBO in suppressing C─H activation, with both scavengers diminishing predefined product selectivity. Furthermore, DMPO‐mediated EPR analysis was employed to identify radical intermediates, whereas TEMPO‐based EPR analysis specifically tracked hole (h^+^). In situ characterization revealed superimposed signals of predominant carbon‐centered and minor oxygen‐centered radicals on the Co_3_S_4_/Sv‐CdIn_2_S_4_/Ti_3_C_2_ HNR surface, visually demonstrating the C─H bond extraction process (Figure 8i; Figure S13). The detected h^+^ and •OH in the system suggests a potential synergistic initiation mechanism for C─H bond activation. During the HMF‐to‐DFF transformation catalyzed by Co_3_S_4_/Sv‐CdIn_2_S_4_/Ti_3_C_2_ HNR, in situ DRIFTS analysis demonstrated that prolonged illumination resulted in progressive attenuation of the C─OH peak at 1092 cm^−1^ with a concomitant intensity enhancement of the C═O peak at 1654 cm^−1^ [40], directly confirming HMF consumption and DFF formation (Figure 8g).

Density functional theory simulations elucidate the atomic‐scale mechanism of PCET‐mediated HMF‐to‐DFF conversion over Co_3_S_4_/Sv‐CdIn_2_S_4_/Ti_3_C_2_ HNR. Computational geometry optimization reveals as‐synthesized catalysts preferentially initiate two consecutive dehydrogenation processes via C_α_‐H bond activation (Figure 6j,k; Figure S14). The Co_3_S_4_/Sv‐CdIn_2_S_4_/Ti_3_C_2_ heterostructure demonstrates superior catalytic performance, characterized by more thermodynamically favorable in terms of exothermic energy (−1.0 eV in Co_3_S_4_/Sv‐CdIn_2_S_4_/Ti_3_C_2_ HNR vs. −0.6 eV in Normal CdIn_2_S_4_) and lower activation barrier of the first hydrogen extraction (kinetically relevant step, 0.96 eV in Co_3_S_4_/Sv‐CdIn_2_S_4_/Ti_3_C_2_ HNR vs. 1.13 eV in Normal CdIn_2_S_4_). Furthermore, electron density difference evidence BIEF‐mediated dual‐interface electron transfer channels, characterized by hole (h^+^) localization in Co_3_S_4_ regions concurrent with electron (e^−^) accumulation on Ti_3_C_2_ surfaces (Figure 6e–g). Bader charge analysis visualizes the proton transport channels: the interfacial lattice oxygen (O^Cd–O–Ti^/O^In–O–Ti^) exhibits significantly enhanced electron density, wherein (i) the electron‐enriched lattice O serves as proton traps to realize HMF molecule nucleophilic abstraction, facilitating C_α_‐H bond cleavage, and (ii) the hydrophilic Ti_3_C_2_ MXene surface establishes a dynamic hydrogen‐bonding nanonetworks to guide rapid proton transfer.

Based on the aforementioned findings, we propose a PCET‐mediated catalytic cycle: (i) Within the electron‐transport channel, an asymmetric dual‐interface BIEFs constructed at the Co_3_S_4_/CdIn_2_S_4_ S‐scheme interface and Sv‐CdIn_2_S_4_/Ti_3_C_2_ Schottky interfaces sharing CdIn_2_S_4_ as a central mediator, drives directional hole migration to the Co_3_S_4_ sector for oxidation reaction and electron migration to the Ti_3_C_2_ MXene for reduction reaction; (ii) Within the proton‐transport channel, electron‐enriched interfacial lattice oxygen functions as proton traps to achieve HMF deprotonation (C─H cleavage) via nucleophilic abstraction, while the hydrophilic Ti_3_C_2_ MXene islands guide rapid proton transfer along modified dynamic hydrogen‐bonding networks, collectively facilitating photocatalyzed redox initiated by C─H activation. This electron‐proton dual‐transfer‐channel catalyst‐induced PCET mechanism enables precise regulation of C─H bond activation at spatially segregated redox‐active sites (Figure S15).

### 2.6 Substrate Scope

**SCHEME 1:**
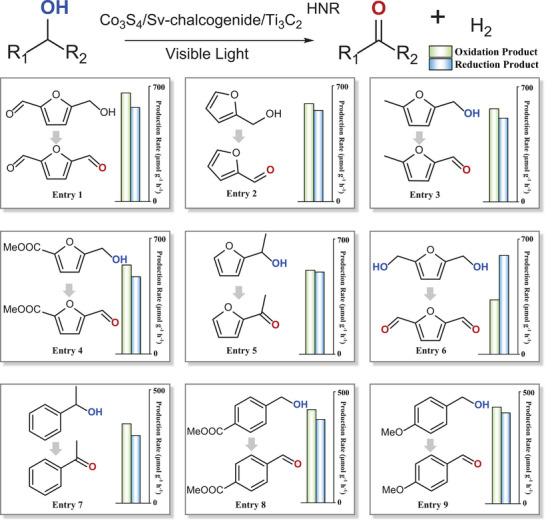
Substrate scope in photocatalytic C─H bond activation performance over Co_3_S_4_/Sv‐chalcogenide/Ti_3_C_2_ HNR.

We systematically investigate the substrate scope of photocatalytic C‐H bond activation reactions within our proton–electron dual‐channel nanoreactor (Scheme 1). The Co_3_S_4_/Sv‐chalcogenide/Ti_3_C_2_ HNR enables precise abstraction of α‐C(sp^3^)‐H and α‐OH protons for C═O bonds formation, maintaining outstanding tolerance toward diverse functional groups. Furan‐derived biomass feedstocks undergo efficient transformation to target products, where electron‐donating/electron‐withdrawing substituted furan derivatives, secondary alcohols, and dual‐reactive‐site substrates all demonstrate high reactivity (Scheme 1 entries 1–6). This C─H activation strategy is extendable to aromatic derivatives, where both electron‐deficient and electron‐rich benzyl alcohol substrates have the opportunity for transforming to corresponding aldehydes, while secondary alcohols undergo conversion to ketones (Scheme 1 entries 7–9). The aldehyde/ketone compounds synthesized via this ternary‐component photocatalysts demonstrate significant potential for implementation across agrochemical, medicinal, and material sciences.

## 3 Conclusion

In summary, we have developed a general proton–electron dual‐transfer‐channel regulation strategy for synthesizing hollow hierarchical Co_3_S_4_/Sv‐chalcogenide/Ti_3_C_2_ nanoreactors (chalcogenide = CdIn_2_S_4_/ZnIn_2_S_4_/CdS) via lateral epitaxy and defect‐mediated heterocomponent anchorage. These architectures integrate dynamic hydrogen‐bonding nanonetworks as proton‐transport channel (via MXene terminal functional groups) and asymmetric dual‐interface BIEFs as electron‐transport channel (via core framework) for realizing PCET in photocatalytic C─H activation. The dual‐interface BIEFs, constructed by the Co_3_S_4_/CdIn_2_S_4_ S‐scheme interface and Sv‐ CdIn_2_S_4_/Ti_3_C_2_ Schottky interface, sharing CdIn_2_S_4_ as a central mediator, not only drives feeble photogenerated electron–hole recombination in Co_3_S_4_ CB and CdIn_2_S_4_ VB while powerful photogenerated hole–electron enrichment in Co_3_S_4_ VB and CdIn_2_S_4_ CB, but also enables steering of photocarrier localization and delocalized‐electron transport from CdIn_2_S_4_ to Ti_3_C_2_ while inhibiting reverse electron flow, thus directing photogenerated holes and electrons transport toward Co_3_S_4_ and CdIn_2_S_4_. Meanwhile, the dynamic hydrogen‐bonding nanonetworks, constructed by the interaction between hydrophilic terminal groups (─O/─OH) on MXene surfaces and water molecules, leverage the electron‐rich interfacial lattice oxygen to mediate the substrate deprotonation process, delivering protons to Ti_3_C_2_ and forming Ti_3_C_2_(OH)^+^ intermediates, thus guiding directional proton transfer along ordered hydration layers. The PCET mechanism facilitated by these proton–electron dual‐channel photocatalysts achieve superior C─H activation efficiency through precisely regulated intermediate adsorption/activation, bypassing high‐energy intermediates. Notably, representative Co_3_S_4_/Sv‐CdIn_2_S_4_/Ti_3_C_2_ HNR exhibits excellent productivity (659 µmol·g^−1^·h^−1^ oxidation product, 571 µmol·g^−1^·h^−1^ reduction product) and maintain potential in biomass‐derived reagents (nine examples). This work establishes a pioneering paradigm for manipulating proton–electron dual‐transfer channels in customized multifunctional site catalysts, offering inspiration for multifunctional photocatalysts capable of addressing complex reaction pathway challenges in sustainable chemical synthesis.

## Author Contributions

Y.F., N.Y., and X.B.L. conceived and supervised the project. Y.W.H., Y.F., and N.Y. wrote the manuscript. Y.W.H., Y.F., X.B.L., and L.Y. designed the synthesis and mechanism experiments. R.Y.L., Y.C., and T.J.G. performed the synthesis and mechanism experiments. All the authors participated in the discussion and improvement of the manuscript.

## Conflicts of Interest

The authors declare no conflicts of interest.

## Supporting information




**Supporting File**: advs74462‐sup‐0001‐SuppMat.pdf

## Data Availability

The data that supports the findings of this study are available in the supplementary material of this article.
