# Supplementary material for: Dynamic Hydrogen‐Bonding Nanonetworks and Asymmetric Dual‐Interface Built‐In Electric Fields Cooperatively Mediate Proton‐Coupled Electron Transfer for C─H Activation
Source: Adv Sci (Weinh). 2026 Mar 30;13(24):e74462. doi: 10.1002/advs.74462 (PMC13116130; doi:10.1002/advs.74462)
Supplement: Supplementary file 1 — Supporting File: advs74462‐sup‐0001‐SuppMat.pdf [file ADVS-13-e74462-s001.pdf]

Supporting Information

Dynamic Hydrogen-Bonding Nanonetworks and Asymmetric Dual-Interface Built-in Electric Fields Cooperatively Mediate Proton-Coupled Electron Transfer for C-H Activation

Yi-Wen Han[a,b‡*], Run-Yu Liu[c‡], Yu Chen[c], Lei Ye[c], Tian-Jun Gong[a*], Xue-Bin Lu[c*], Ning Yan[b,d*], Yao Fu[a*]

[a]State Key Laboratory of Precision and Intelligent Chemistry, Anhui Province Key Laboratory of Biomass Chemistry, University of Science and Technology of China, 230026, Hefei, China.

[b]Department of Chemical and Biomolecular Engineering, National University of Singapore, 4 Engineering Drive 4, 117585, Singapore.

[c]School of Environmental Science and Engineering, Tianjin University, 300350, Tianjin, China.

[d]Centre for Hydrogen Innovations, National University of Singapore, 1 Engineering Drive 3, 117580, Singapore.

[*]Corresponding Author. E-mail: ywhanustc@mail.ustc.edu.cn; gongtj@ustc.edu.cn; xbltju@tju.edu.cn; ning.yan@nus.edu.sg; fuyao@ustc.edu.cn

Experimental details

1 Materials

Chemicals: All starting materials are purchased from Aladdin and Macklin unless otherwise noted and used without further purification.

2 Catalyst Preparation

Synthesis of Hollow Co_3S_4 Nanocage (Co_3S_4 HNC)

Using as-prepared ZIF-67 as a precursor, ortho-dodecahedral **Co_3S_4 HNC** are synthesized. 8 mmol of $Co(NO_3)_2·6H_2O$ and 16 mmol of 2-methylimidazole are dissolved in methanol. The above two solutions are mixed under magnetic stirring. The mixture solution is aged for 24 hours. The sample is collected by centrifugation and washed with deionized (DI) water and ethanol several times, and then dried in the vacuum oven. The obtained samples are labeled as **ZIF-67**. The sulfur source is added afterward for etching. The resulting ZIF-67 and thioacetamide (TAA) are dispersed in ethanol. Afterward, the mixed solution is transferred to a Teflon-lined autoclave, heated at 120 °C for 4 hours. The sample is collected by centrifugation and washed with DI water and ethanol several times, and then dried in the vacuum oven. The obtained samples are labeled as **Co_3S_4 HNC**.

Synthesis of Hollow $Co_3S_4/CdIn_2S_4$ Nanoreactor ($Co_3S_4/CdIn_2S_4$ HNR)

The **$Co_3S_4/CdIn_2S_4$ HNR** are synthesized via an opportune lateral epitaxy method. 8 mL glycerol is added to HCl aqueous solution, and then **Co_3S_4** is added and stirred for 30 min. $CdCl_2$ (366 mg), $InCl_3$ (586 mg), and TAA (300 mg) are added and stirred for 30 min. Afterward, the mixed solution is transferred to an oil bath and stirred at 80°C for 4 hours. The sample is collected by centrifugation and washed with DI water and ethanol several times, and then dried in the vacuum oven. The obtained samples are labeled as **$Co_3S_4/CdIn_2S_4$ HNR**. Furthermore, **$Co_3S_4/ZnIn_2S_4$ HNR** and **Co_3S_4/CdS HNR** are synthesized using the same lateral epitaxy method. When $ZnCl_2$, $InCl_3$, and TAA are used as synthetic precursors, **$Co_3S_4/ZnIn_2S_4$ HNR** is obtained. When $CdCl_2$ and TAA are used as synthetic precursors, **Co_3S_4/CdS HNR** is obtained.

Synthesis of Ti_3C_2 MXene

The **Ti_3C_2 MXene** are synthesized by modified literature methods.[1,2]

Synthesis of Hollow Co_3S_4/Sv-$CdIn_2S_4$/Ti_3C_2 Nanoreactor (Co_3S_4/Sv-$CdIn_2S_4$/Ti_3C_2 HNR)

Co_3S_4/Sv-$CdIn_2S_4$ (sulfur-vacancy Co_3S_4/$CdIn_2S_4$) is prepared with a modified chemical reduction method, and hollow **Co_3S_4/Sv-$CdIn_2S_4$/Ti_3C_2** nanoreactor (**Co_3S_4/Sv-$CdIn_2S_4$/Ti_3C_2 HNR**) is fabricated by defect-mediated heterocomponent anchorage strategy according to improved literature methods.[3,4] The typical process includes as-synthesized sample addition, ultrasonication, centrifugation, washing, and drying. The **Co_3S_4/Sv-$CdIn_2S_4$/Ti_3C_2** HNR with different mass percentages of Ti_3C_2 (3%, 6%, 9%) is denoted as **Co_3S_4/Sv-$CdIn_2S_4$/Ti_3C_2-1 HNR, Co_3S_4/Sv-$CdIn_2S_4$/Ti_3C_2-2 HNR, Co_3S_4/Sv-$CdIn_2S_4$/Ti_3C_2-3 HNR,** respectively. The **Co_3S_4/Sv-$ZnIn_2S_4$/Ti_3C_2-2 HNR** and **Co_3S_4/Sv-CdS/Ti_3C_2-2 HNR** are synthesized by a similar process to that of **Co_3S_4/Sv-$CdIn_2S_4$/Ti_3C_2-2 HNR**.

Synthesis of Normal $CdIn_2S_4$

Normal $CdIn_2S_4$ are synthesized according to a modified hydrothermal method. Typically, 4.5 mmol TAA is dissolved in DI water under vigorous stirring for 30 min. Then, 1.5 mmol $Cd(NO_3)_2·4H_2O$ and 2.2 mmol $In(NO_3)_2·4H_2O$ are added into the solution, and the mixture is continuously stirred for another 30 min. Afterward, the mixed solution is transferred into a Teflon-lined autoclave and heated at 180 °C for 20 h. After the reaction, the sample is collected by centrifugation and washed with DI water and ethanol several times, and then dried in the vacuum oven. The samples obtained are labeled as **Normal $CdIn_2S_4$**.

Synthesis of Sv-$CdIn_2S_4$/Ti_3C_2

Sv-$CdIn_2S_4$/Ti_3C_2 are synthesized according to a modified literature method.[5]

Synthesis of Co_3S_4 & Sv-$CdIn_2S_4$ & Ti_3C_2 HNR

Co_3S_4 & Sv-$CdIn_2S_4$ & Ti_3C_2 are prepared by a simple physical mixing method. Typically, the as-synthesized **Co_3S_4 HNR**, **Sv-$CdIn_2S_4$** and **Ti_3C_2** powders are ground together in an agate mortar for 10 min to ensure uniform mixing. The obtained composite powders are denoted as **Co_3S_4 & Sv-$CdIn_2S_4$ & Ti_3C_2 HNR.**

3 Material Characterizations

The intrinsic properties of the as-prepared samples are investigated by the following characterizations. Scanning electron microscopy (SEM, Hitachi SU8010), transmission electron microscopy (TEM, JEM-200CX) are employed to examine morphology and size. Energy-dispersive spectroscopy (EDS) and X-ray photoelectron spectroscopy (XPS, ULVAC-PHI5000) are used to measure the chemical compositions. N_2 adsorption/desorption measurements are carried out to study Brunauer-Emmett-Teller (BET, Micrometritics, ASAP 2460) surface areas and pore volume. Crystallographic structure is conducted by X-ray diffraction (XRD, Bruker D8 Advance Diffractometer) with monochromatic Cu Kα radiation. Ultraviolet-visible diffuse reflectance spectra (UV-vis DRS, Shimadzu UV 3600) is used to evaluate light absorption ability. Raman spectra is measured on Renishaw inVia. AFM (Bruker) with a Kelvin probe force microscopy (KPFM) mode is conducted to measure the surface potential of samples. The surface photovoltage (SPV) spectra is tested by a Bruker dimension V scanning probe microscope equipped with a lock-in amplifier. XPS and darkness/irradiation XPS are used to clarify the direction of charge migration. Photoluminescence spectra are used to characterize steady state photoluminescence (PL) and time-resolved photoluminescence (TRPL). Electrochemical experiments are conducted on CHI-660E electrochemical workstation (Chenhua) with a standard three-electrode system. Femtosecond transient absorption spectroscopy (fs-TAS) is used to examine the dynamics of carriers via a pump-probe experimental program. The electron paramagnetic resonance tests (EPR, JEOL, JES-FA200) are performed with 5,5-dimethyl-1-pyrroline-N-oxide-(DMPO) and 2,2,6,6-tetramethylpiperidinyloxy-(TEMPO) as spin-trap agents. In-situ DRIFTS measurements are performed on a Thermo Nicolet iS50 spectrometer equipped with an MCT detector under light irradiation to monitor the evolution of surface species. The X-ray absorption spectroscopy data is collected at the BL14W1 station in the Shanghai Synchrotron Radiation Facility.

4 Photocatalytic C-H Activation and Corresponding Products Analysis

Assessment of the photocatalytic activity of the samples is carried out under the irradiation of a xenon lamp with a UV-cut filter. Typically, 10 mg catalyst and 10 mL HMF reaction solution (10 mM) are incorporated into a sealed photochemical reactor. Before reaction, the air in the reaction system is removed. The liquid product is

analyzed by high-performance liquid chromatography (HPLC, Shimadzu) and gaseous product is analyzed by gas chromatography (GC, Agilent). The apparent quantum efficiency (AQE) is calculated based on the following equation:

$$AQE = \frac{N_e}{N_p} \times 100\% = \frac{2 \times M \times N_A \times h \times c}{S \times P \times t \times \lambda} \times 100\%$$

where Ne is the amount of reaction electrons, Np is the incident photons, N_A is Avogadro constant, h is the Planck constant, c is the speed of light, S is the irradiation area, P is the intensity of the irradiation, t is the photoreaction time, and λ is the wavelength of the monochromatic light.

Substrate scope: 10 mg photocatalyst, 5 mM substrates, 10 mL reaction solution.

5 The Through-Plane Proton Conductivity Analysis

The through-plane proton conductivity of the as-synthesized catalysts is measured using a two-electrode AC impedance spectroscopy technique. The proton conductivities (σ, mS cm^{-1}) of as-synthesized catalysts are calculated based on the following equation:

$$\sigma = \frac{I}{R \times A}$$

where I (cm), R (Ω), and A (cm^2) represent the thickness, resistance, and contact area, respectively.

6 Density Functional Theory (DFT) Calculations

We have employed the Vienna Ab Initio Package (VASP)[6,7] to perform all the density functional theory (DFT) calculations within the generalized gradient approximation (GGA) using the PBE[8] formulation. We have chosen the projected augmented wave (PAW) potentials[9] to describe the ionic cores and take valence electrons into account using a plane wave basis set with a kinetic energy cutoff of 400 eV. Partial occupancies of the Kohn−Sham orbitals are allowed using the Gaussian smearing method and a width of 0.05 eV. Electronic energy is considered self-consistent when the energy change is smaller than 10^{-5} eV. Geometry optimization is considered convergent when the force change is smaller than 0.02 eV/Å. Grimme's DFT-D3 methodology is used to describe the dispersion interactions.

Model 1 is a $CdIn_2S_4$ surface model. The equilibrium lattice constants of $CdIn_2S_4$ unit cell are optimized. We then use it to construct a $CdIn_2S_4$ surface model with $p(1\times 1)$ periodicity in the X and Y directions and 1 stoichiometric layer in the Z direction separated by a vacuum layer in the depth of 15 Å in order to separate the surface slab

from its periodic duplicates. During structural optimizations, a 2×2×1 k-point grid in the Brillouin zone is used for k-point sampling, and all atoms are allowed to relax. Other models derived from $CdIn_2S_4$ are obtained by changing the parameters based on the mentioned $CdIn_2S_4$ model.

Model 2 is a MXene surface model. The equilibrium lattice constants of MXene unit cell are optimized. We then use it to construct a MXene surface with a $p(1\times\sqrt{3})$ periodicity in the X and Y and 1 stoichiometric layer in the Z direction separated by a Z-direction vacuum layer in the depth of 15 Å in order to separate the surface slab from its periodic duplicates. During structural optimizations, a 4×6×1 k-point grid in the Brillouin zone is used for k-point sampling, and all atoms are allowed to relax. Other models derived from MXene are obtained by changing the parameters based on the mentioned MXene model.

Model 3 is a Co_3S_4 surface model. The equilibrium lattice constants of Co_3S_4 unit cell are optimized. We then use it to construct a Co_3S_4 surface model with $p(1\times1)$ periodicity in the X and Y directions and 1 stoichiometric layer in the Z direction separated by a vacuum layer in the depth of 15 Å in order to separate the surface slab from its periodic duplicates. During structural optimizations, the 2×2×1 k-point mesh in the Brillouin zone is used for k-point sampling, and all atoms are allowed to relax. Other models derived from Co_3S_4 are obtained by changing the parameters based on the mentioned Co_3S_4 model.

Model 4 is a $Co_3S_4/CdIn_2S_4$ heterojunction surface model. The Co_3S_4 part has a $p(1\times1)$ periodicity in the X and Y directions and 1 stoichiometric layer in the Z direction, the $CdIn_2S_4$ part has a $p(1\times1)$ periodicity in the X and Y directions and 1 stoichiometric layer in the Z direction, the whole slab is separated by a vacuum layer in the depth of 15 Å in order to separate the surface slab from its periodic duplicates. During structural optimizations, a 2×2×1 k-point grid in the Brillouin zone is used for k-point sampling, and the bottom half of $CdIn_2S_4$ part is fixed while all the rest are allowed to relax. Other models derived from $Co_3S_4/CdIn_2S_4$ are obtained by changing the parameters based on the mentioned $Co_3S_4/CdIn_2S_4$ model.

Model 5 is a $CdIn_2S_4$/MXene heterojunction surface model. The $CdIn_2S_4$ part has a $p(1\times2)$ periodicity in the X and Y directions and 1 stoichiometric layer in the Z direction, the MXene part has a $p(2\sqrt{3}\times7)$ periodicity in the X and Y directions and 1 stoichiometric layer in the Z direction, the whole slab is separated by a vacuum layer in the depth of 15 Å in order to separate the surface slab from its periodic duplicates.

During structural optimizations, the gamma point in the Brillouin zone is used for k-point sampling, and the bottom half of MXene part is fixed while all the rest are allowed to relax. Other models derived from $CdIn_2S_4$/MXene are obtained by changing the parameters based on the mentioned $CdIn_2S_4$/MXene model.

The transition state of an elementary reaction step is located by the nudged elastic band (NEB) method. In the NEB method, the path between the reactant(s) and product(s) is discretized into a series of five structural images. The intermediate images are relaxed until the perpendicular forces are smaller than 0.02 eV/Å.

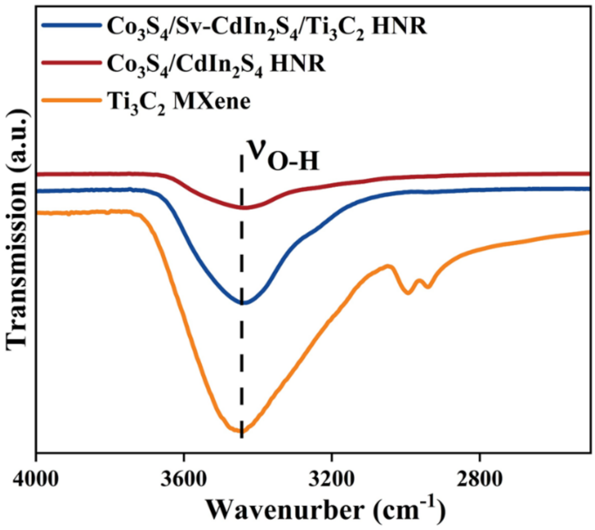

Figure S1. FTIR spectra of Co_3S_4/Sv-$CdIn_2S_4$/Ti_3C_2 HNR, Co_3S_4/$CdIn_2S_4$ HNR, and Ti_3C_2 MXene, highlighting surface hydroxyl groups.

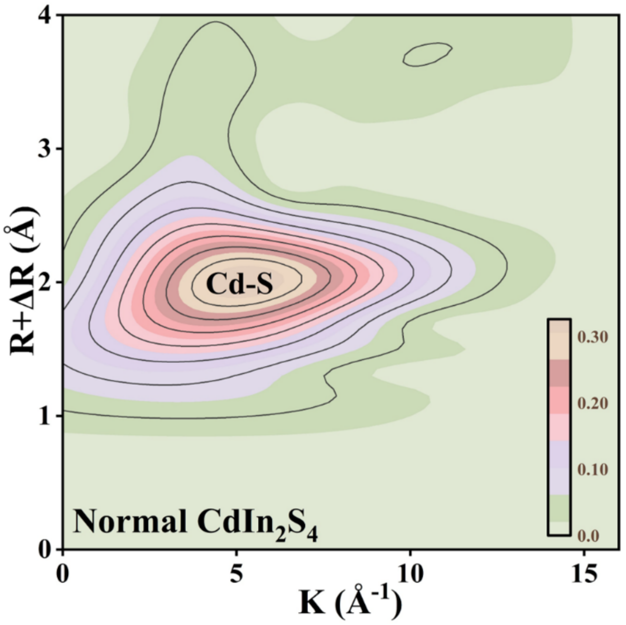

Figure S2. Wavelet transform (WT) analysis of Cd-K edge for Normal $CdIn_2S_4$.

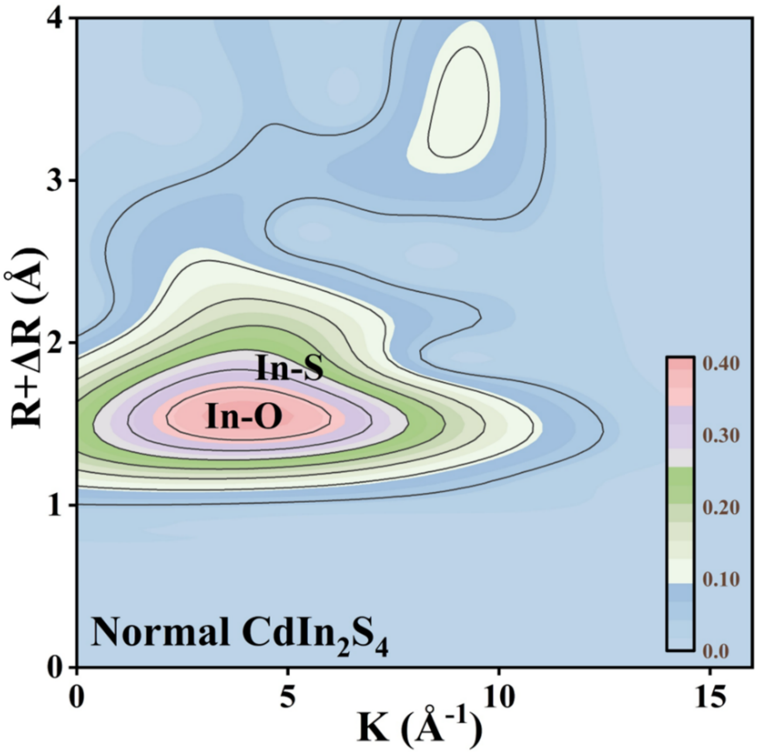

Figure S3. Wavelet transform (WT) analysis of In-K edge for Normal $CdIn_2S_4$.

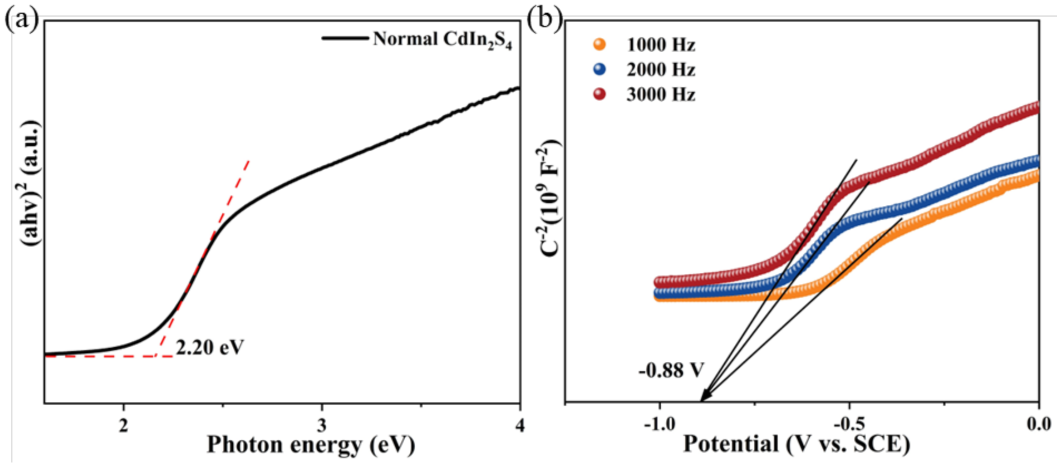

Figure S4. a) Tauc's plot of Normal $CdIn_2S_4$, b) Mott-Schottky plot of Normal $CdIn_2S_4$.

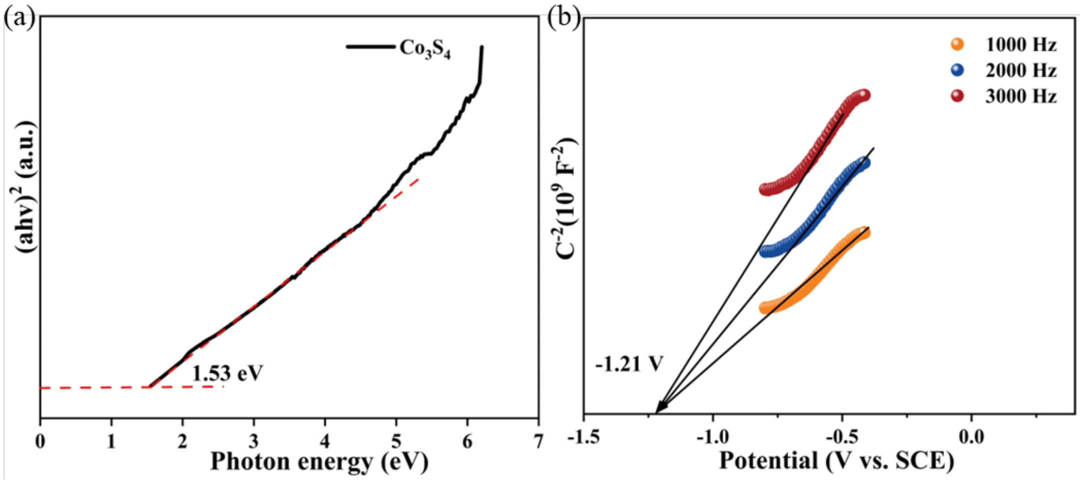

Figure S5. a) Tauc's plot of Co_3S_4 HNR, b) Mott-Schottky plot of Co_3S_4 HNR.

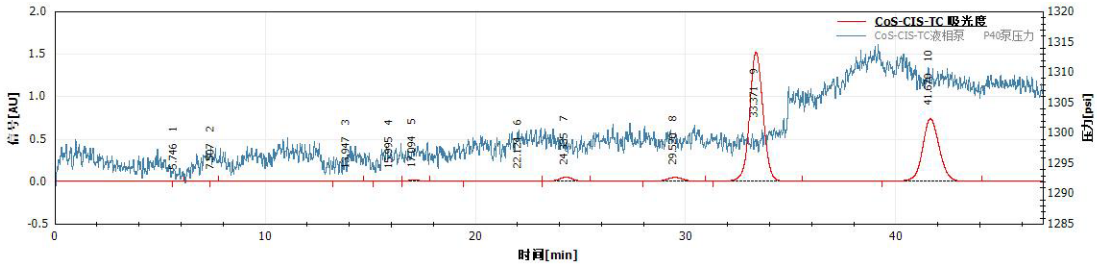

Figure S6. The original HPLC of representative Co_3S_4/Sv-$CdIn_2S_4$/Ti_3C_2 HNR.

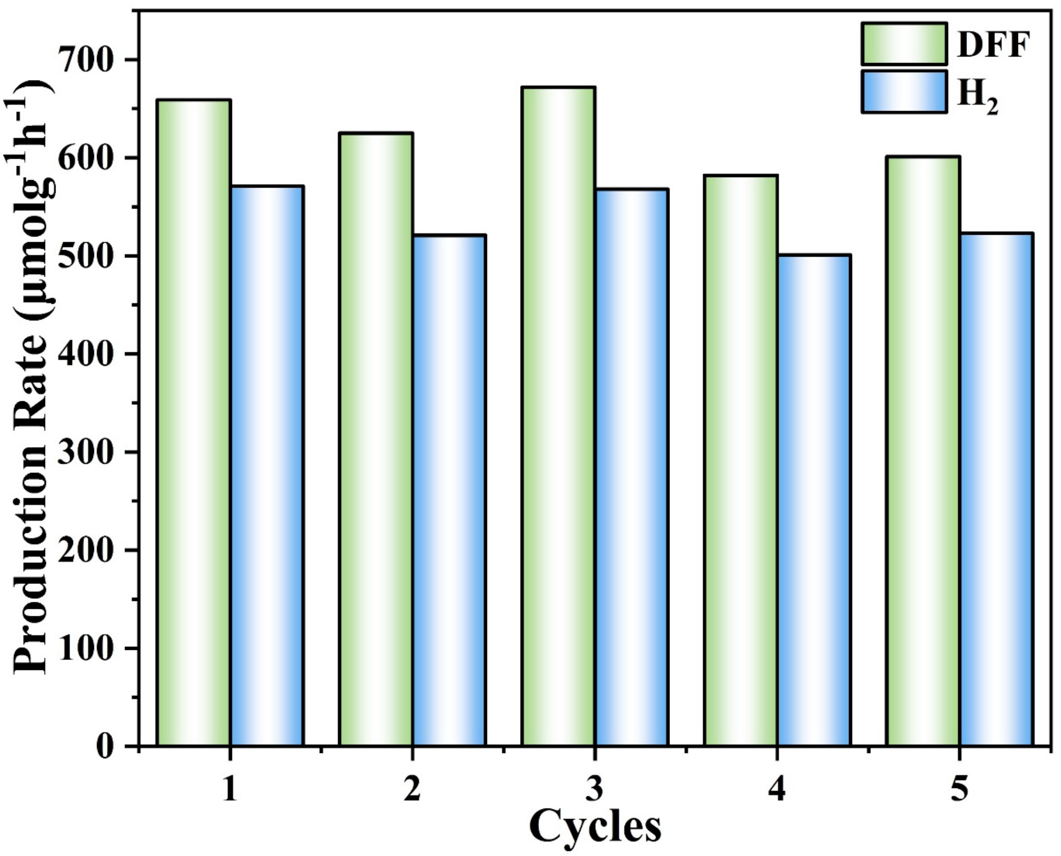

Figure S7. Cyclability testing of Co_3S_4/Sv-$CdIn_2S_4$/Ti_3C_2 HNR.

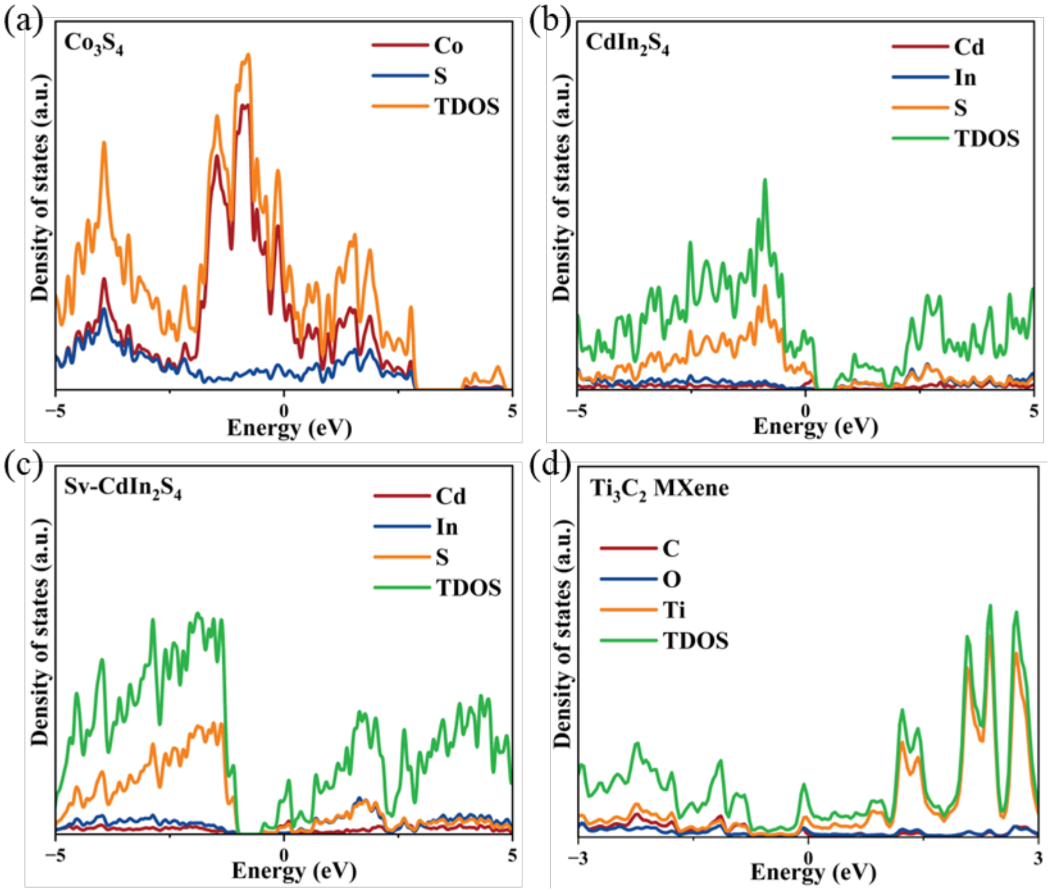

Figure S8. Calculated density of states (DOS) of a) Co_3S_4, b) $CdIn_2S_4$, c) Sv-$CdIn_2S_4$, d) Ti_3C_2 MXene.

As shown in DOS, Ti_3C_2 MXene is fully filled with electrons, implying its metallic property and outstanding electrical conductivity. Meanwhile, there are significant band gaps in Co_3S_4 and $CdIn_2S_4$. On the other hand, based on the average electrostatic potential along the Z-axis, the simulated work functions (Φ) of Co_3S_4, $CdIn_2S_4$, Sv-$CdIn_2S_4$, Ti_3C_2 are separately 5.57, 5.23, 5.07, 6.08 eV, suggesting electrons on $CdIn_2S_4$ can transfer to Co_3S_4 in the Co_3S_4/$CdIn_2S_4$ S-scheme heterojunction, electrons on Sv-$CdIn_2S_4$ can transfer to Ti_3C_2 in the Sv-$CdIn_2S_4$/Ti_3C_2 Schottky heterojunction. Theoretically, when Co_3S_4, $CdIn_2S_4$, and Ti_3C_2 nanoparticles are in close contact within the ordered nanostructure (Co_3S_4 core - $CdIn_2S_4$ shell - surface immobilization Ti_3C_2), under illumination, holes will accumulate at the VB of Co_3S_4 and electrons will accumulate on Ti_3C_2, thereby generating strong redox capabilities.

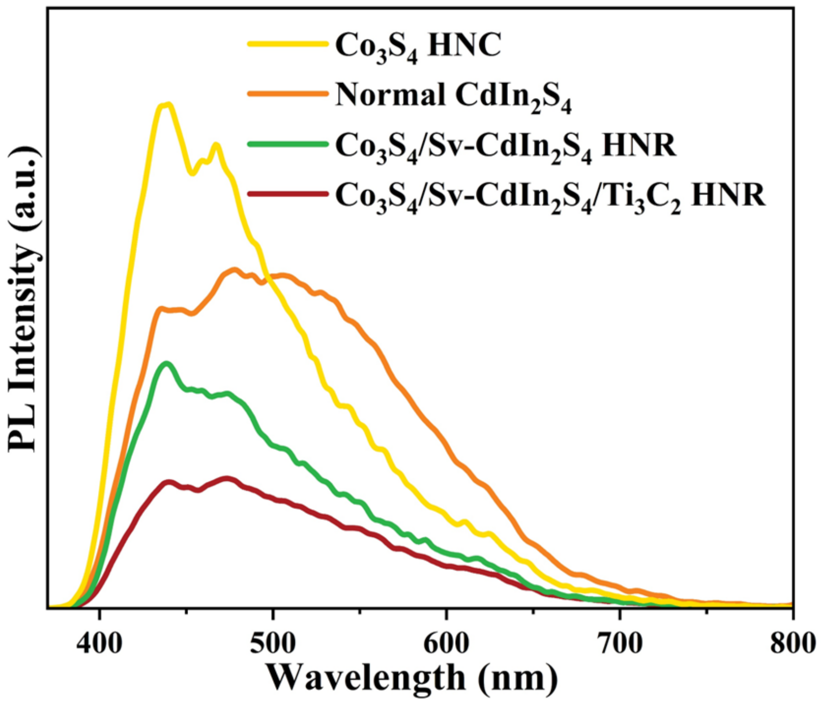

Figure S9. The PL spectra of Co_3S_4 HNR, Normal $CdIn_2S_4$, Co_3S_4/Sv-$CdIn_2S_4$ HNR, and Co_3S_4/Sv-$CdIn_2S_4$/Ti_3C_2 HNR.

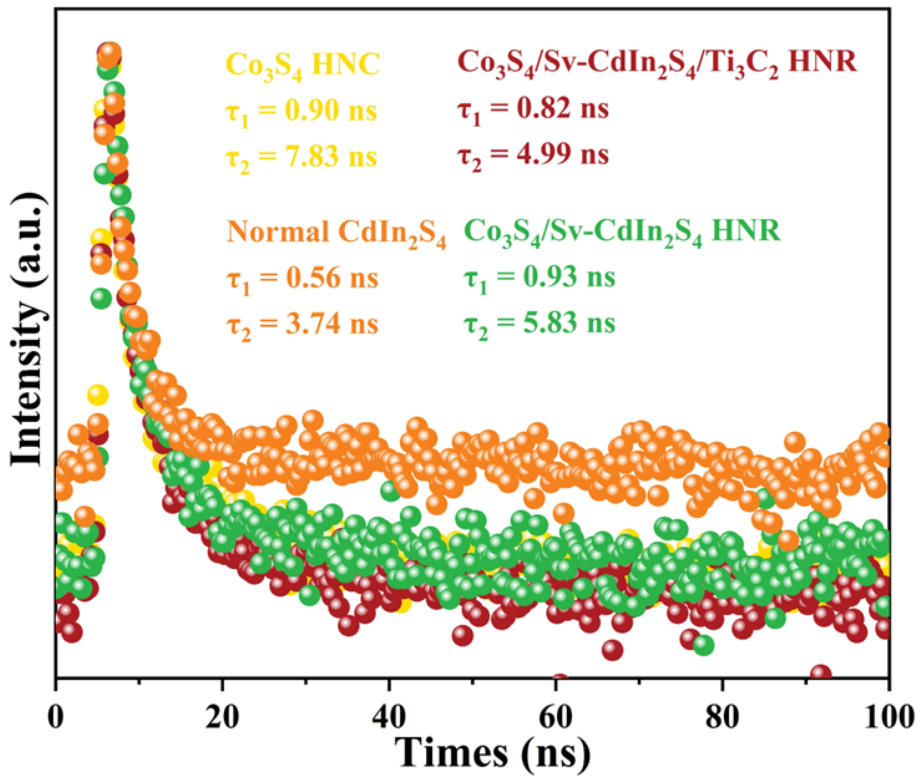

Figure S10. The time-resolved PL spectra of Co_3S_4 HNC, Normal $CdIn_2S_4$, Co_3S_4/Sv-$CdIn_2S_4$ HNR, and Co_3S_4/Sv-$CdIn_2S_4$/Ti_3C_2 HNR.

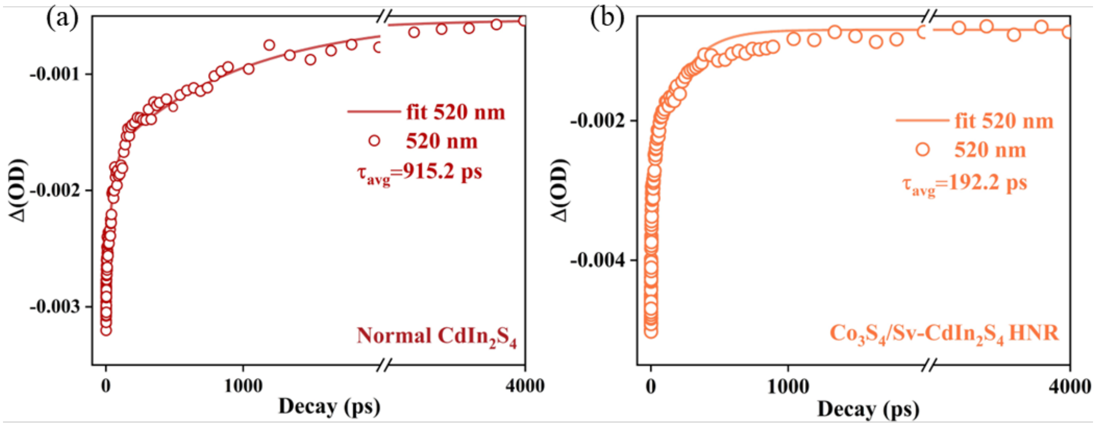

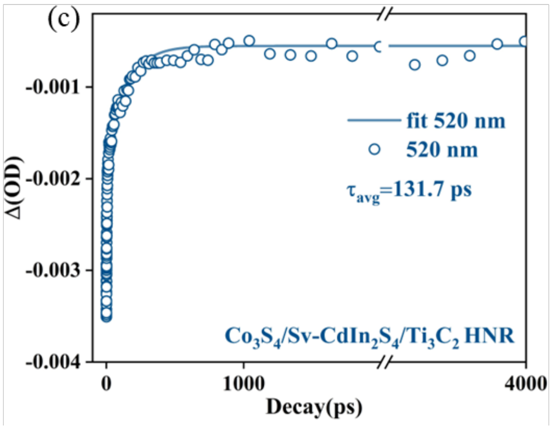

Figure S11. The decay signals of Normal $CdIn_2S_4$, Co_3S_4/Sv-$CdIn_2S_4$ HNR, and Co_3S_4/Sv-$CdIn_2S_4$/Ti_3C_2 HNR.

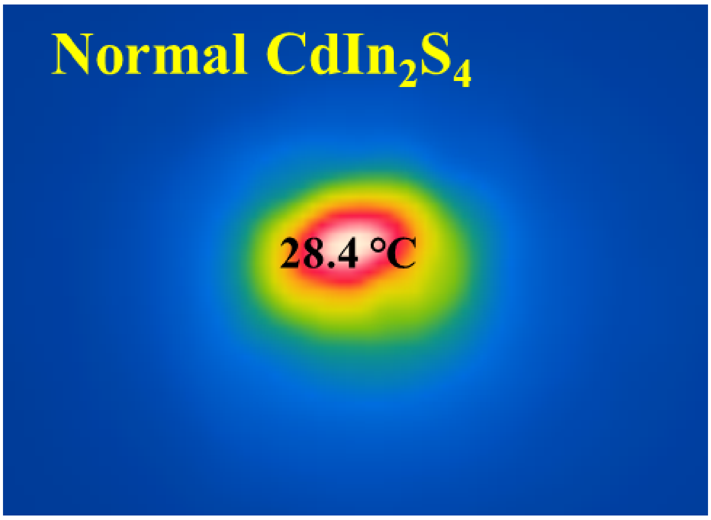

Figure S12. Surface temperature of Normal $CdIn_2S_4$.

Surface temperature measurements demonstrate localized surface plasmon resonance (LSPR)-induced photothermal conversion in the Ti_3C_2 MXene component of the ternary heterostructure: plasmon-driven transformation of concentrated photogenerated electrons into long-lived hot electrons enhances the photothermal effect during relaxation (127.2 °C for Co_3S_4/Sv-$CdIn_2S_4$/Ti_3C_2 HNR vs. 28.4 °C for Normal $CdIn_2S_4$).[10,11]

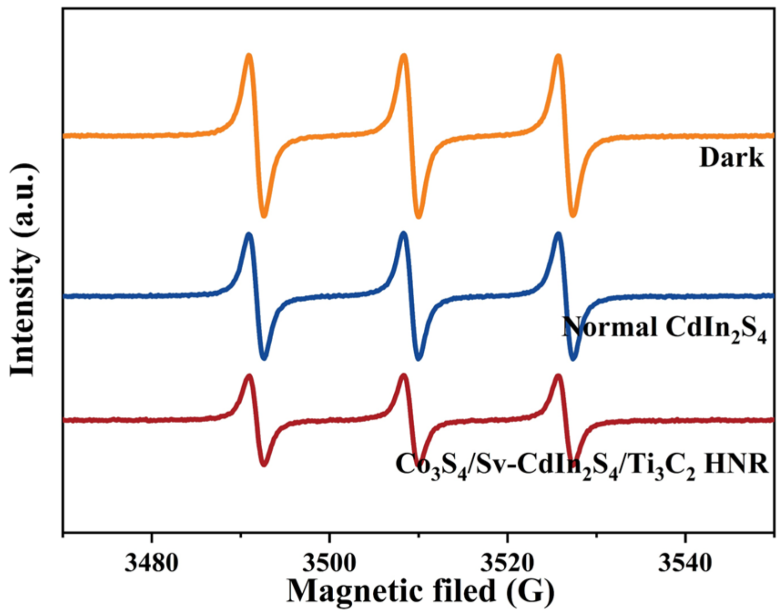

Figure S13. EPR measurement over as-prepared photocatalysts.

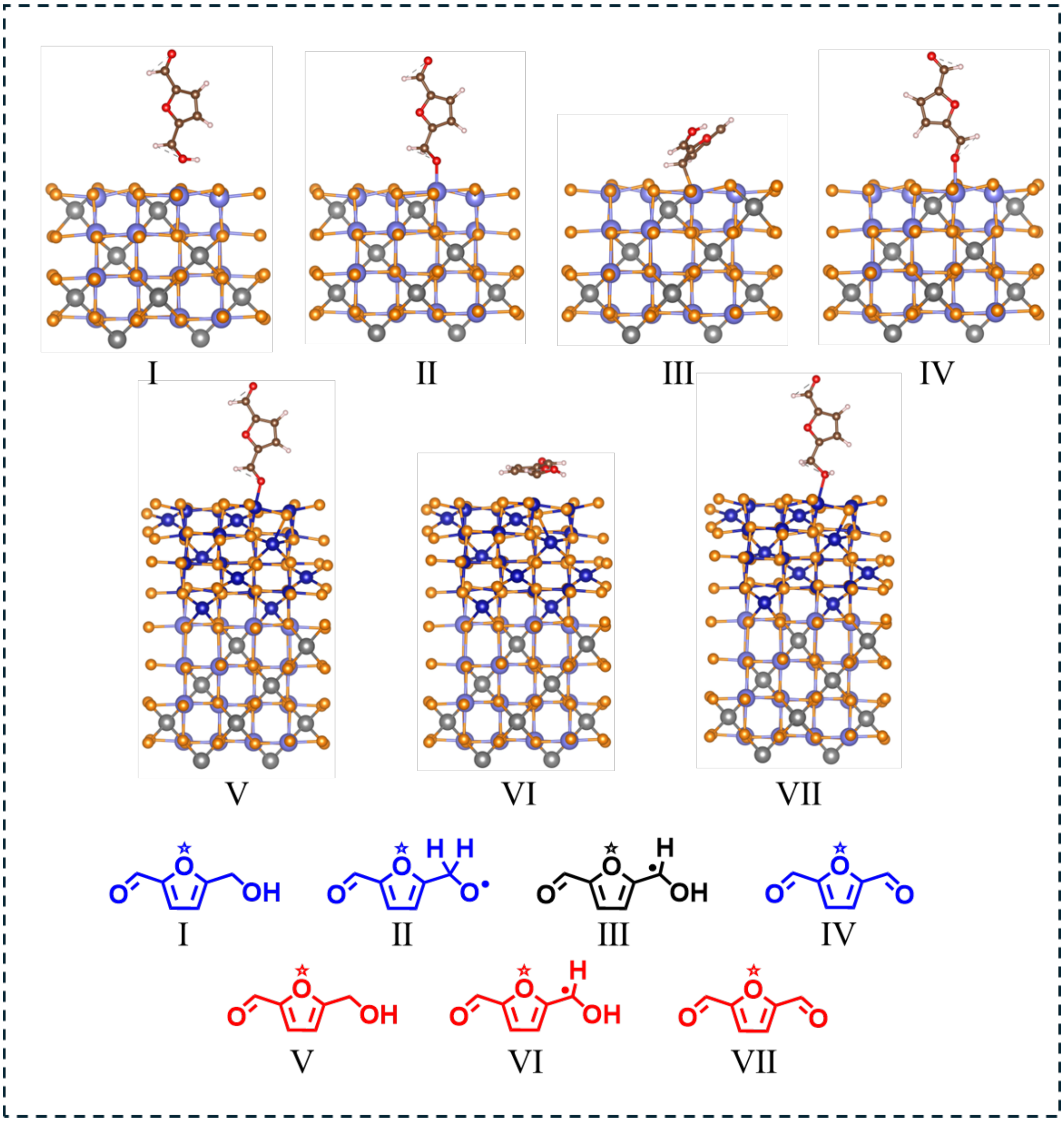

Figure S14. DFT optimized structures on as-synthesized photocatalysts.

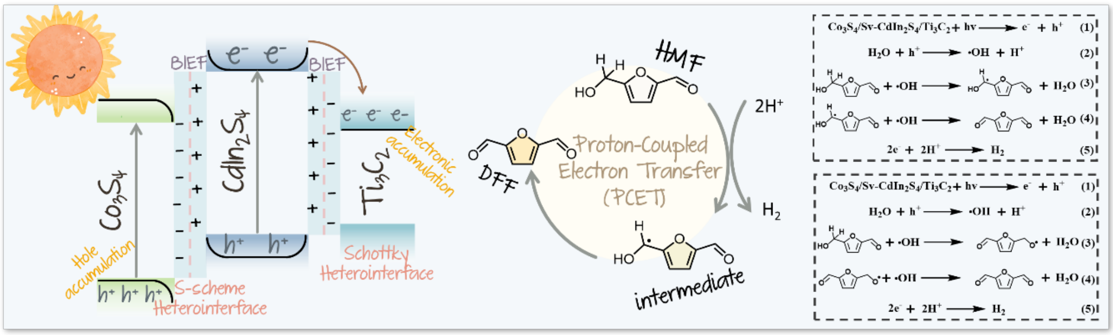

Figure S15. Possible mechanism of photocatalytic selective HMF photooxidation coupled with H_2 generation over hollow Co_3S_4/Sv-$CdIn_2S_4$/Ti_3C_2 dual-transfer-channel nanoreactor via a PCET pathway.

Table S1. EXAFS fitting parameters at the Cd K-edge for as-prepared samples.

Sample	Shell	CN[a]	R^b(Å)	σ^{2c}(Å^2)	$\Delta E_0{}^d$(eV)	R factor
Normal $CdIn_2S_4$	Cd-S	5.9±0.2	2.60±0.01	0.0125	-1.4±0.5	0.0033
Co_3S_4/Sv-$CdIn_2S_4$ /Ti_3C_2 HNR	Cd-S	5.5±0.3	2.62±0.02	0.0110	-1.0±0.9	0.0152
	Cd-O	0.4±0.2	2.19±0.02	0.0030		

[a]*CN*: coordination numbers; [b]*R*: bond length; [c]σ^2: Debye-Waller factors; [d] ΔE_0: the inner potential correction. R factor: goodness of the fitting.

Table S2. EXAFS fitting parameters at the In K-edge for as-prepared samples.

Sample	Shell	CN[a]	R^b(Å)	σ^{2c}($Å^2$)	$\Delta E_0{}^d$(eV)	R factor
Normal $CdIn_2S_4$	In-S	3.0±0.4	2.53±0.02	0.0131	1.0±0.8	0.0080
Co_3S_4/Sv-$CdIn_2S_4$	In-S	2.4±0.2	2.60±0.02	0.0080	3.4±0.5	0.0038
/Ti_3C_2 HNR	In-O	3.4±0.2	2.14±0.02	0.0055		

[a]*CN*: coordination numbers; [b]*R*: bond length; [c]σ^2: Debye-Waller factors; [d] ΔE_0: the inner potential correction. R factor: goodness of the fitting.

Atomic Coordinates in DFT Calculation

I

10.9200000763	0.0000000000	0.0000000000
0.0000000000	10.9200000763	0.0000000000
0.0000000000	0.0000000000	24.6562995911

H	C	O	Cd	In	S
6	6	3	8	16	32

Direct

0.815271974	0.070294999	0.650569975
0.736522019	0.127434000	0.558165014
0.452024013	0.196431994	0.789641023
0.794494987	0.064616002	0.761498988
0.592377007	0.273000985	0.590210974
0.487305999	0.149297997	0.595911980
0.738022029	0.099944003	0.675018013
0.629190981	0.147021994	0.655739009
0.612815976	0.141284004	0.744997025
0.727400005	0.096464999	0.732222974
0.546611011	0.159053996	0.795085013
0.584542990	0.174335003	0.599882007
0.552016973	0.172463998	0.697462976
0.588149011	0.135462001	0.840429008
0.649711013	0.102242999	0.560777009
0.250000000	0.250000000	0.081119999
0.750000000	0.750000000	0.081119999
0.000000000	0.500000000	0.191839993
0.500000000	0.000000000	0.191839993
0.747983992	0.248045996	0.302767992
0.249525994	0.749518991	0.302038997

0.499527991	0.500322998	0.422307998
0.002410000	0.997861028	0.422735989
0.875000000	0.125000000	0.136480004
0.625000000	0.375000000	0.136480004
0.375000000	0.625000000	0.136480004
0.125000000	0.875000000	0.136480004
0.375000000	0.375000000	0.247199997
0.125000000	0.125000000	0.247199997
0.625000000	0.625000000	0.247199997
0.875000000	0.875000000	0.247199997
0.127964005	0.370483994	0.363083005
0.869867027	0.630681992	0.363056988
0.370449007	0.128496006	0.363498986
0.628423989	0.871465981	0.362699986
0.369911999	0.867018998	0.457897007
0.625935972	0.126147002	0.461609006
0.867624998	0.370584995	0.456705004
0.130049005	0.629988015	0.457868010
0.634280026	0.134279996	0.132369995
0.865719974	0.365720004	0.132369995
0.134279996	0.634280026	0.132369995
0.365720004	0.865719974	0.132369995
0.384279996	0.384279996	0.140589997
0.615719974	0.615719974	0.140589997
0.115719996	0.115719996	0.140589997
0.884280026	0.884280026	0.140589997
0.884280026	0.115719996	0.243090004
0.615719974	0.384279996	0.243090004
0.384279996	0.615719974	0.243090004

0.115719996	0.884280026	0.243090004
0.365720004	0.134279996	0.251309991
0.865719974	0.634280026	0.251309991
0.634280026	0.865719974	0.251309991
0.134279996	0.365720004	0.251309991
0.630797029	0.631274998	0.353960007
0.868651986	0.869459987	0.354236007
0.367599994	0.368129998	0.354499996
0.130944997	0.131165996	0.354360014
0.112144001	0.612815976	0.354748011
0.611896992	0.112976998	0.356546998
0.886191010	0.387535989	0.353928000
0.385728002	0.886668980	0.354728013
0.611624002	0.883167982	0.465640992
0.109881997	0.388482988	0.466904998
0.384905994	0.109240003	0.467494011
0.887170970	0.612262011	0.466717988
0.360727996	0.636892021	0.480093002
0.138725996	0.860041976	0.480455995
0.638217986	0.361467004	0.478873014
0.863314986	0.139477998	0.478451997

II

10.9200000763	0.0000000000	0.0000000000
0.0000000000	10.9200000763	0.0000000000
0.0000000000	0.0000000000	24.6562995911

H	C	O	Cd	In	S
5	6	3	8	16	32

Direct

0.798974991	0.111602001	0.640824974
0.425514013	0.154376999	0.784355998
0.788111985	0.108401000	0.752795994
0.541328013	0.249271005	0.581543028
0.456034005	0.114061996	0.592139006
0.719763994	0.122694999	0.666966021
0.601329029	0.140938997	0.648630023
0.591979027	0.137162998	0.738120019
0.713685989	0.120621003	0.724084020
0.526574016	0.141306996	0.788868010
0.551567972	0.150279999	0.592510998
0.522671998	0.149505004	0.691253006
0.574899971	0.131057993	0.833975971
0.627645016	0.087963000	0.557008028
0.250000000	0.250000000	0.081119999
0.750000000	0.750000000	0.081119999
0.000000000	0.500000000	0.191839993
0.500000000	0.000000000	0.191839993
0.743997991	0.243993998	0.303662986
0.248464003	0.748470008	0.302336007
0.501215994	0.498405010	0.422073990
0.000782000	0.999387980	0.421415001
0.875000000	0.125000000	0.136480004
0.625000000	0.375000000	0.136480004
0.375000000	0.625000000	0.136480004
0.125000000	0.875000000	0.136480004
0.375000000	0.375000000	0.247199997
0.125000000	0.125000000	0.247199997
0.625000000	0.625000000	0.247199997

0.875000000	0.875000000	0.247199997
0.127341002	0.368144989	0.362356007
0.868196011	0.627287984	0.362323999
0.374426007	0.129113004	0.363397002
0.629052997	0.873524010	0.363151997
0.366681010	0.865926981	0.456425995
0.627236009	0.122042999	0.472041011
0.866294026	0.366946012	0.456279993
0.127826005	0.627291977	0.457192004
0.634280026	0.134279996	0.132369995
0.865719974	0.365720004	0.132369995
0.134279996	0.634280026	0.132369995
0.365720004	0.865719974	0.132369995
0.384279996	0.384279996	0.140589997
0.615719974	0.615719974	0.140589997
0.115719996	0.115719996	0.140589997
0.884280026	0.884280026	0.140589997
0.884280026	0.115719996	0.243090004
0.615719974	0.384279996	0.243090004
0.384279996	0.615719974	0.243090004
0.115719996	0.884280026	0.243090004
0.365720004	0.134279996	0.251309991
0.865719974	0.634280026	0.251309991
0.634280026	0.865719974	0.251309991
0.134279996	0.365720004	0.251309991
0.629948020	0.631605029	0.353742987
0.868570030	0.867062986	0.354124010
0.367505014	0.368120998	0.354319990
0.132751003	0.129994005	0.353715986

0.110820003	0.610585988	0.354184002
0.614623010	0.113890998	0.362403989
0.885223985	0.385322988	0.353318006
0.386193991	0.886313975	0.353469014
0.607308984	0.875566006	0.466111004
0.109415002	0.385964006	0.466371000
0.379655004	0.105593003	0.466547996
0.886027992	0.608807981	0.466282010
0.358566999	0.635007024	0.478684992
0.136069998	0.857483029	0.478902996
0.638235986	0.362964988	0.480076998
0.867091000	0.138818994	0.478709996

III

10.9200000763	0.0000000000	0.0000000000
0.0000000000	10.9200000763	0.0000000000
0.0000000000	0.0000000000	24.6562995911

H	C	O	Cd	In	S
5	6	3	8	16	32

Direct

0.466304988	0.389324009	0.535341978
0.698951006	0.137089998	0.639854014
0.861644983	0.479528010	0.656086028
0.606800020	0.592481971	0.548985004
0.489928991	0.140789002	0.570019007
0.566263020	0.386696994	0.539870024
0.613071024	0.291161001	0.577902973
0.709874988	0.464628011	0.598975003
0.624602020	0.499350995	0.562389016

0.797725022	0.536047995	0.630958974
0.571754992	0.177640006	0.589396000
0.712746024	0.337985992	0.607158005
0.801402986	0.648087978	0.631061971
0.623335004	0.100469001	0.626075029
0.250000000	0.250000000	0.081119999
0.750000000	0.750000000	0.081119999
0.000000000	0.500000000	0.191839993
0.500000000	0.000000000	0.191839993
0.749871016	0.249782994	0.302017003
0.248766005	0.749538004	0.302047998
0.480035007	0.519101024	0.415520012
0.998296022	0.001283000	0.424190015
0.875000000	0.125000000	0.136480004
0.625000000	0.375000000	0.136480004
0.375000000	0.625000000	0.136480004
0.125000000	0.875000000	0.136480004
0.375000000	0.375000000	0.247199997
0.125000000	0.125000000	0.247199997
0.625000000	0.625000000	0.247199997
0.875000000	0.875000000	0.247199997
0.129785001	0.370965987	0.362646013
0.871688008	0.628629029	0.364823014
0.369825006	0.126822993	0.363341987
0.628355026	0.869229019	0.362798005
0.368164003	0.871863008	0.459073007
0.628129005	0.111928999	0.458297014
0.889174998	0.370000988	0.459677011
0.126981005	0.629566014	0.459764987

0.634280026	0.134279996	0.132369995
0.865719974	0.365720004	0.132369995
0.134279996	0.634280026	0.132369995
0.365720004	0.865719974	0.132369995
0.384279996	0.384279996	0.140589997
0.615719974	0.615719974	0.140589997
0.115719996	0.115719996	0.140589997
0.884280026	0.884280026	0.140589997
0.884280026	0.115719996	0.243090004
0.615719974	0.384279996	0.243090004
0.384279996	0.615719974	0.243090004
0.115719996	0.884280026	0.243090004
0.365720004	0.134279996	0.251309991
0.865719974	0.634280026	0.251309991
0.634280026	0.865719974	0.251309991
0.134279996	0.365720004	0.251309991
0.632084012	0.631111979	0.353267998
0.868475020	0.868001997	0.355134994
0.367830992	0.367235005	0.352982998
0.130236000	0.131003007	0.354707986
0.112875000	0.612999022	0.356070012
0.612520993	0.112282000	0.355067015
0.885622978	0.386321008	0.356303006
0.386036992	0.885369003	0.355513990
0.613506019	0.876573980	0.466412991
0.125120997	0.384723991	0.466533005
0.388747990	0.112750001	0.467186004
0.885469973	0.606189013	0.469090998
0.358900011	0.640257001	0.482710987

0.136123002	0.862133980	0.480278999
0.624705970	0.365900993	0.467352003
0.855220973	0.144611001	0.481774986

IV

10.9200000763	0.0000000000	0.0000000000
0.0000000000	10.9200000763	0.0000000000
0.0000000000	0.0000000000	24.6562995911

H	C	O	Cd	In	S
4	6	3	8	16	32

Direct

0.394160002	0.138530001	0.612399995
0.668179989	0.125469998	0.796159983
0.335810006	0.138359994	0.721229970
0.765929997	0.128240004	0.609239995
0.455440015	0.135869995	0.647080004
0.582170010	0.132090002	0.643899977
0.535920024	0.131860003	0.730809987
0.425949991	0.135800004	0.702690005
0.567539990	0.130050004	0.788030028
0.666760027	0.131209999	0.598320007
0.632070005	0.129500002	0.694930017
0.491869986	0.133220002	0.824959993
0.632089972	0.133709997	0.550999999
0.250000000	0.250000000	0.081119999
0.750000000	0.750000000	0.081119999
0.000000000	0.500000000	0.191839993
0.500000000	0.000000000	0.191839993
0.749509990	0.249540001	0.302049994

0.249610007	0.749549985	0.302040011
0.497799993	0.497909993	0.424369991
0.999710023	0.999689996	0.422580004
0.875000000	0.125000000	0.136480004
0.625000000	0.375000000	0.136480004
0.375000000	0.625000000	0.136480004
0.125000000	0.875000000	0.136480004
0.375000000	0.375000000	0.247199997
0.125000000	0.125000000	0.247199997
0.625000000	0.625000000	0.247199997
0.875000000	0.875000000	0.247199997
0.128729999	0.371239990	0.363330007
0.868900001	0.628870010	0.363000005
0.371280015	0.128720000	0.363330007
0.628970027	0.868910015	0.363050014
0.369280010	0.868200004	0.457470000
0.632799983	0.128230006	0.458079994
0.867950022	0.369179994	0.457480013
0.128209993	0.632929981	0.458000004
0.634280026	0.134279996	0.132369995
0.865719974	0.365720004	0.132369995
0.134279996	0.634280026	0.132369995
0.365720004	0.865719974	0.132369995
0.384279996	0.384279996	0.140589997
0.615719974	0.615719974	0.140589997
0.115719996	0.115719996	0.140589997
0.884280026	0.884280026	0.140589997
0.884280026	0.115719996	0.243090004
0.615719974	0.384279996	0.243090004

0.384279996	0.615719974	0.243090004
0.115719996	0.884280026	0.243090004
0.365720004	0.134279996	0.251309991
0.865719974	0.634280026	0.251309991
0.634280026	0.865719974	0.251309991
0.134279996	0.365720004	0.251309991
0.629740000	0.629750013	0.354409993
0.868449986	0.868279994	0.354130000
0.368169993	0.368149996	0.354900002
0.131650001	0.131630003	0.354600012
0.112300001	0.613080025	0.354970008
0.613210022	0.112219997	0.355010003
0.886659980	0.386970013	0.354420006
0.387070000	0.886690021	0.354420006
0.613860011	0.885450006	0.466690004
0.106859997	0.390700012	0.467200011
0.390579998	0.107100002	0.467189997
0.885389984	0.613829970	0.466639996
0.360500008	0.640470028	0.480040014
0.137820005	0.862349987	0.480120003
0.640200019	0.360529989	0.480080009
0.862309992	0.137759998	0.480120003

V

10.0670003891	0.0000000000	0.0000000000
0.0000000000	10.0670003891	0.0000000000
0.0000000000	0.0000000000	35.0000000000

H	C	O	Cd	In	S	Co
6	6	3	8	16	64	24

Direct

0.846924007	0.181923002	0.711314976
0.726529002	0.058552001	0.652170002
0.440331012	0.106328003	0.810898006
0.833625019	0.162015006	0.789691985
0.518637002	0.240656003	0.670266986
0.501845002	0.065218002	0.673584998
0.759594023	0.164796993	0.728855014
0.631560028	0.149615005	0.715654016
0.621271014	0.135056004	0.778536022
0.752810001	0.155037001	0.769240975
0.550019026	0.119658999	0.814227998
0.572876990	0.148241997	0.676581979
0.546173990	0.130954996	0.745276988
0.603466988	0.121105999	0.845862985
0.676234007	0.140728995	0.648239970
0.250000000	0.250000000	0.065719999
0.750000000	0.750000000	0.065719999
0.000000000	0.500000000	0.143720001
0.500000000	0.000000000	0.143720001
0.750000000	0.250000000	0.221719995
0.250000000	0.750000000	0.221719995
0.500728011	0.499307990	0.303849012
0.999401987	0.000633000	0.303867996
0.875000000	0.125000000	0.104719996
0.625000000	0.375000000	0.104719996
0.375000000	0.625000000	0.104719996
0.125000000	0.875000000	0.104719996
0.375000000	0.375000000	0.182720006

0.125000000	0.125000000	0.182720006
0.625000000	0.625000000	0.182720006
0.875000000	0.875000000	0.182720006
0.125000000	0.375000000	0.260720015
0.875000000	0.625000000	0.260720015
0.375000000	0.125000000	0.260720015
0.625000000	0.875000000	0.260720015
0.379022986	0.879064023	0.343984991
0.619408011	0.119246997	0.343652010
0.880280972	0.380549014	0.343811989
0.119726002	0.619608998	0.344377995
0.634280026	0.134279996	0.101820000
0.865719974	0.365720004	0.101820000
0.134279996	0.634280026	0.101820000
0.365720004	0.865719974	0.101820000
0.384279996	0.384279996	0.107610002
0.615719974	0.615719974	0.107610002
0.115719996	0.115719996	0.107610002
0.884280026	0.884280026	0.107610002
0.884280026	0.115719996	0.179820001
0.615719974	0.384279996	0.179820001
0.384279996	0.615719974	0.179820001
0.115719996	0.884280026	0.179820001
0.365720004	0.134279996	0.185609996
0.865719974	0.634280026	0.185609996
0.634280026	0.865719974	0.185609996
0.134279996	0.365720004	0.185609996
0.634280026	0.634280026	0.257820010
0.865719974	0.865719974	0.257820010

0.365720004	0.365720004	0.257820010
0.134279996	0.134279996	0.257820010
0.115719996	0.615719974	0.263610005
0.615719974	0.115719996	0.263610005
0.884280026	0.384279996	0.263610005
0.384279996	0.884280026	0.263610005
0.631869018	0.865005016	0.340770006
0.134552002	0.367015988	0.340797007
0.365085006	0.131787002	0.340698004
0.867115974	0.634644985	0.340824991
0.368407995	0.630333006	0.349169999
0.130325004	0.868291974	0.349225014
0.630701005	0.368699014	0.349860013
0.868806005	0.131001994	0.349763006
0.639006019	0.139084995	0.418565005
0.860406995	0.360377997	0.418686002
0.141500995	0.641062975	0.422859013
0.355962008	0.855841994	0.422722012
0.391348988	0.385113001	0.410645992
0.612856984	0.607840002	0.410620004
0.108001001	0.112868004	0.410690993
0.885353029	0.891608000	0.410719991
0.883974016	0.113734998	0.476788014
0.614301026	0.383473009	0.477025002
0.404442996	0.593387008	0.474007994
0.094062001	0.904052973	0.474103987
0.355881989	0.140318006	0.470995009
0.855211020	0.646456003	0.470797002
0.640582979	0.855287015	0.471055001

0.147187993	0.354819000	0.470984995
0.637808979	0.641380012	0.537423015
0.858991027	0.854740024	0.537500024
0.355641007	0.358749986	0.537524998
0.142532006	0.138198003	0.537491977
0.123287000	0.622734010	0.522256970
0.594483018	0.095206998	0.529021978
0.904609978	0.404415011	0.524221003
0.374478996	0.873634994	0.522437990
0.586001992	0.901404023	0.595999002
0.089010000	0.411341995	0.595988989
0.403324008	0.085643999	0.595851004
0.911013007	0.589430988	0.595766008
0.354669005	0.641152024	0.587704003
0.141828999	0.854731023	0.587211013
0.646216989	0.353338987	0.579630017
0.851188004	0.148892999	0.575169981
0.249907002	0.249081999	0.381312996
0.749099016	0.749827981	0.381386995
0.871971011	0.127418995	0.415358007
0.627228975	0.372076005	0.415385008
0.383197010	0.614714980	0.413244992
0.115295000	0.883105993	0.413334996
0.998004019	0.497498989	0.445874006
0.500093997	0.999836028	0.443944991
0.379213005	0.366138995	0.473818988
0.121918000	0.129851997	0.473838001
0.630116999	0.621035993	0.473787010
0.866496980	0.878677011	0.473874986

0.751748025	0.248538002	0.514369011
0.249415994	0.748718023	0.485267013
0.891390979	0.626320004	0.532585979
0.370490015	0.105976000	0.533006012
0.126216993	0.390316010	0.532855988
0.606038988	0.870060980	0.532998979
0.495106995	0.500975013	0.563099980
0.002053000	0.994728982	0.562489986
0.624598980	0.122837000	0.588234007
0.368532985	0.868054986	0.581448972
0.872438014	0.373376012	0.583252013
0.128858998	0.628814995	0.581211984

VI

10.0670003891	0.0000000000	0.0000000000
0.0000000000	10.0670003891	0.0000000000
0.0000000000	0.0000000000	35.0000000000

H	C	O	Cd	In	S	Co
5	6	3	8	16	64	24

Direct

0.389055997	0.262380004	0.658115983
0.835363984	0.122769997	0.653500021
0.838604987	0.555328012	0.677998006
0.438861012	0.526278019	0.669073999
0.567534029	0.037581000	0.662482023
0.484838009	0.310342997	0.661643982
0.608677030	0.246350005	0.659956992
0.647058010	0.460826010	0.669161022
0.510044992	0.445576012	0.667039990

0.729762971	0.575675011	0.676546991
0.644119024	0.109315000	0.654483020
0.707876980	0.337965012	0.665264010
0.683547020	0.687986016	0.681190014
0.767152011	0.064332001	0.664479017
0.250000000	0.250000000	0.065719999
0.750000000	0.750000000	0.065719999
0.000000000	0.500000000	0.143720001
0.500000000	0.000000000	0.143720001
0.750000000	0.250000000	0.221719995
0.250000000	0.750000000	0.221719995
0.499859005	0.500147998	0.303880006
0.999041021	0.000905000	0.303894997
0.875000000	0.125000000	0.104719996
0.625000000	0.375000000	0.104719996
0.375000000	0.625000000	0.104719996
0.125000000	0.875000000	0.104719996
0.375000000	0.375000000	0.182720006
0.125000000	0.125000000	0.182720006
0.625000000	0.625000000	0.182720006
0.875000000	0.875000000	0.182720006
0.125000000	0.375000000	0.260720015
0.875000000	0.625000000	0.260720015
0.375000000	0.125000000	0.260720015
0.625000000	0.875000000	0.260720015
0.378962994	0.880191982	0.344110012
0.619165003	0.119971000	0.343640000
0.880945027	0.380515993	0.343715996
0.119709998	0.620350003	0.344435006

0.634280026	0.134279996	0.101820000
0.865719974	0.365720004	0.101820000
0.134279996	0.634280026	0.101820000
0.365720004	0.865719974	0.101820000
0.384279996	0.384279996	0.107610002
0.615719974	0.615719974	0.107610002
0.115719996	0.115719996	0.107610002
0.884280026	0.884280026	0.107610002
0.884280026	0.115719996	0.179820001
0.615719974	0.384279996	0.179820001
0.384279996	0.615719974	0.179820001
0.115719996	0.884280026	0.179820001
0.365720004	0.134279996	0.185609996
0.865719974	0.634280026	0.185609996
0.634280026	0.865719974	0.185609996
0.134279996	0.365720004	0.185609996
0.634280026	0.634280026	0.257820010
0.865719974	0.865719974	0.257820010
0.365720004	0.365720004	0.257820010
0.134279996	0.134279996	0.257820010
0.115719996	0.615719974	0.263610005
0.615719974	0.115719996	0.263610005
0.884280026	0.384279996	0.263610005
0.384279996	0.884280026	0.263610005
0.631650984	0.865729988	0.340604991
0.134997994	0.367763996	0.340920001
0.364895999	0.132896006	0.341019988
0.867136002	0.634911001	0.340826005
0.368478000	0.631492972	0.349332988

0.130338997	0.869111001	0.349276990
0.631424010	0.369331986	0.349417001
0.868864000	0.130961001	0.350125015
0.638888001	0.140190005	0.418563008
0.859878004	0.361427009	0.418570012
0.140787005	0.642768979	0.422964990
0.356418997	0.857869983	0.422814012
0.392459989	0.386685997	0.410737008
0.613031030	0.609228015	0.410685986
0.107794002	0.113568999	0.410735011
0.885151029	0.891700029	0.410654008
0.882499993	0.116143003	0.477322996
0.611792982	0.385870010	0.476307988
0.403847992	0.596032023	0.474198997
0.093502000	0.906131983	0.474103987
0.354409993	0.142764002	0.471635014
0.854303002	0.649639010	0.470642000
0.640625000	0.857756972	0.471284986
0.145942003	0.356581002	0.470685989
0.638696015	0.644481003	0.537395000
0.858968019	0.857701004	0.537647009
0.353296012	0.359268010	0.537433028
0.140524000	0.138714001	0.537688971
0.121766999	0.624674976	0.522131026
0.595175982	0.096652001	0.529527009
0.902822018	0.406639993	0.522971988
0.373780012	0.876097023	0.522390008
0.584716976	0.904314995	0.595779002
0.086736001	0.412907004	0.595476985

0.398613989	0.085766003	0.596387982
0.911617994	0.588777006	0.595197022
0.354577988	0.642318010	0.587116003
0.141057998	0.854837000	0.588168979
0.648028016	0.350201994	0.572816014
0.854970992	0.146598995	0.582165003
0.249678999	0.250007004	0.381718010
0.749271989	0.750992000	0.381197989
0.872192025	0.127765998	0.415628999
0.627547026	0.372536987	0.414934993
0.383531004	0.616747975	0.413410991
0.114542998	0.884011984	0.413347989
0.995984018	0.499610990	0.445811987
0.499756008	0.002515000	0.444507986
0.377269000	0.368770003	0.473859012
0.120270997	0.131663993	0.473879009
0.629721999	0.624664009	0.473686010
0.866267025	0.882023990	0.473894000
0.743261993	0.256197006	0.514190972
0.248539999	0.750966012	0.485529989
0.889572978	0.628073990	0.532352984
0.369529009	0.109989002	0.534169972
0.124242999	0.392565012	0.532227993
0.607500017	0.872949004	0.533738017
0.494765013	0.502992988	0.561830997
0.000047000	0.995998979	0.563874006
0.619341016	0.130046993	0.592337012
0.368065000	0.869669020	0.581331015
0.870333016	0.371672988	0.581709027

0.128907993	0.629953980	0.581148982

VII

10.0670003891	0.0000000000	0.0000000000
0.0000000000	10.0670003891	0.0000000000
0.0000000000	0.0000000000	35.0000000000

H	C	O	Cd	In	S	Co
4	6	3	8	16	64	24

Direct

0.861652017	0.156834006	0.708231986
0.441956013	0.133959994	0.806068003
0.839087009	0.160218999	0.786674023
0.484317988	0.111741997	0.669689000
0.772181988	0.151344001	0.725503981
0.643849015	0.139706001	0.711095989
0.626145005	0.142579004	0.773837984
0.760603011	0.152774006	0.765500009
0.552040994	0.140883997	0.809505999
0.591821015	0.131155998	0.673142016
0.554225028	0.133998007	0.740629971
0.605220020	0.146959007	0.841093004
0.666307986	0.145217001	0.644686997
0.250000000	0.250000000	0.065719999
0.750000000	0.750000000	0.065719999
0.000000000	0.500000000	0.143720001
0.500000000	0.000000000	0.143720001
0.750000000	0.250000000	0.221719995
0.250000000	0.750000000	0.221719995
0.500692010	0.499307990	0.303862005

0.999471009	0.000595000	0.303855985
0.875000000	0.125000000	0.104719996
0.625000000	0.375000000	0.104719996
0.375000000	0.625000000	0.104719996
0.125000000	0.875000000	0.104719996
0.375000000	0.375000000	0.182720006
0.125000000	0.125000000	0.182720006
0.625000000	0.625000000	0.182720006
0.875000000	0.875000000	0.182720006
0.125000000	0.375000000	0.260720015
0.875000000	0.625000000	0.260720015
0.375000000	0.125000000	0.260720015
0.625000000	0.875000000	0.260720015
0.379168004	0.878913999	0.343964010
0.619436979	0.119326003	0.343618006
0.880414009	0.380751997	0.343829989
0.119498000	0.619813025	0.344410002
0.634280026	0.134279996	0.101820000
0.865719974	0.365720004	0.101820000
0.134279996	0.634280026	0.101820000
0.365720004	0.865719974	0.101820000
0.384279996	0.384279996	0.107610002
0.615719974	0.615719974	0.107610002
0.115719996	0.115719996	0.107610002
0.884280026	0.884280026	0.107610002
0.884280026	0.115719996	0.179820001
0.615719974	0.384279996	0.179820001
0.384279996	0.615719974	0.179820001
0.115719996	0.884280026	0.179820001

0.365720004	0.134279996	0.185609996
0.865719974	0.634280026	0.185609996
0.634280026	0.865719974	0.185609996
0.134279996	0.365720004	0.185609996
0.634280026	0.634280026	0.257820010
0.865719974	0.865719974	0.257820010
0.365720004	0.365720004	0.257820010
0.134279996	0.134279996	0.257820010
0.115719996	0.615719974	0.263610005
0.615719974	0.115719996	0.263610005
0.884280026	0.384279996	0.263610005
0.384279996	0.884280026	0.263610005
0.631940007	0.865059972	0.340820998
0.134565994	0.367087007	0.340779990
0.365099013	0.131684005	0.340644002
0.866990983	0.634918988	0.340894997
0.368189991	0.630208015	0.349177003
0.130524993	0.868559003	0.349256992
0.630873978	0.368822992	0.349813014
0.868829012	0.131321996	0.349653989
0.639280975	0.139661998	0.418529987
0.860602021	0.360826999	0.418716013
0.141209006	0.641174018	0.422933012
0.355803013	0.856154025	0.422701001
0.391229004	0.385048002	0.410690993
0.612464011	0.607972980	0.410580009
0.108170003	0.113194004	0.410771996
0.885352015	0.892275989	0.410674006
0.884060979	0.113677002	0.476696998

0.613951981	0.384003997	0.476891994
0.404199004	0.593874991	0.473987013
0.094042003	0.904384971	0.474106014
0.356736004	0.140391007	0.471226990
0.854492009	0.647495985	0.470687002
0.640075982	0.856325984	0.471206993
0.147620007	0.354631990	0.471056998
0.637239993	0.641951978	0.537299991
0.858417988	0.855485976	0.537481010
0.355396003	0.358788013	0.537530005
0.142321005	0.139018998	0.537563026
0.122823000	0.623012006	0.522170007
0.594766021	0.095376000	0.530359983
0.905008972	0.405045003	0.523869991
0.373961985	0.873973012	0.522535980
0.584185004	0.900656998	0.596104026
0.088958003	0.412247986	0.596027017
0.401466012	0.085520998	0.596202016
0.911010027	0.590377986	0.595614016
0.354133010	0.641382992	0.587773979
0.140950993	0.855054975	0.587122977
0.646308005	0.356002003	0.579752982
0.850942016	0.150556996	0.574759007
0.250102997	0.249053001	0.381267011
0.748960018	0.750046015	0.381491989
0.871994972	0.127983004	0.415284991
0.627273977	0.372480005	0.415280998
0.382997006	0.614791989	0.413248986
0.115304001	0.883387983	0.413350999

0.997766972	0.497815996	0.446330994
0.500159979	0.000162000	0.443583012
0.379543006	0.366398990	0.473845989
0.122409001	0.129853994	0.473865986
0.629648983	0.621702015	0.473738015
0.866280019	0.879105985	0.473899007
0.752672970	0.248146996	0.514738977
0.249179006	0.749128997	0.485231996
0.891125023	0.626864016	0.532360017
0.370157987	0.106760003	0.533362985
0.126147002	0.390352011	0.532921016
0.605508029	0.869857013	0.533329010
0.494782001	0.501227021	0.562950015
0.001457000	0.995774984	0.562521994
0.623754025	0.122396000	0.590474010
0.367601991	0.868273020	0.581538975
0.872864008	0.374323994	0.582978010
0.128662005	0.629190981	0.581180990

References

1. Zuo, G., Wang, Y., Teo, W.L., Xie, A., Guo, Y., Dai, Y., Zhou, W., Jana, D., Xian, Q., Dong, W., and Zhao, Y. (2020). Ultrathin $ZnIn_2S_4$ Nanosheets Anchored on $Ti_3C_2T_X$ MXene for Photocatalytic H_2 Evolution. Angew. Chem. Int. Ed. *59*, 11287-11292. 10.1002/anie.202002136.
2. Ran, J., Gao, G., Li, F., Ma, T., Du, A., and Qiao, S. (2017). Ti3C2 MXene co-catalyst on metal sulfide photo-absorbers for enhanced visible-light photocatalytic hydrogen production. Nat. Commun. *8*, 13907. 10.1038/ncomms13907.
3. Su, T., Men, C., Chen, L., Chu, B., Luo, X., Ji, H., Chen, J., and Qin, Z. (2022). Sulfur Vacancy and Ti3C2T Cocatalyst Synergistically Boosting Interfacial Charge Transfer in 2D/2D Ti3C2T/ZnIn2S4 Heterostructure for Enhanced Photocatalytic Hydrogen Evolution. Adv. Sci. *9*, 2103715. https://doi.org/10.1002/advs.202103715.

4. Wang, X., Wang, X., Huang, J., Li, S., Meng, A., and Li, Z. (2021). Interfacial Chemical Bond and Internal Electric Field Modulated Z-scheme S_v-$ZnIn_2S_4/MoSe_2$ Photocatalyst for Efficient Hydrogen Evolution. Nature Commun. *12*, 4112. 10.1038/s41467-021-24511-z.
5. Wenger, O.S. (2013). Proton-Coupled Electron Transfer with Photoexcited Metal Complexes. Acc. Chem. Res. *46*, 1517-1526. 10.1021/ar300289x.
6. Kresse, G., and Furthmüller, J. (1996). Efficiency of ab-initio total energy calculations for metals and semiconductors using a plane-wave basis set. Comp. Mater. Sci. *6*, 15-50. https://doi.org/10.1016/0927-0256(96)00008-0.
7. Kresse, G., and Furthmüller, J. (1996). Efficient Iterative Schemes for Ab Initio Total-energy Calculations Using a Plane-wave Basis Set. Phys. Rev. B *54*, 11169-11186. 10.1103/PhysRevB.54.11169.
8. Perdew, J.P., Burke, K., and Ernzerhof, M. (1996). Generalized Gradient Approximation Made Simple. Phys. Rev. Lett. *77*, 3865-3868. 10.1103/PhysRevLett.77.3865.
9. Kresse, G., and Joubert, D. (1999). From ultrasoft pseudopotentials to the projector augmented-wave method. Phys. Rev. B. *59*, 1758-1775. 10.1103/PhysRevB.59.1758.
10. Zhu, L., Tian, L., Jiang, S., Han, L., Liang, Y., Li, Q., and Chen, S. (2023). Advances in Photothermal Regulation Strategies: From Efficient Solar Heating to Daytime Passive Cooling. Chem. Soc. Rev. *52*, 7389-7460. 10.1039/D3CS00500C.
11. Guo, Y., Sun, J., Tang, Y., Jia, X., Nie, Y., Geng, Z., Wang, C., Zhang, J., Tan, X., Zhong, D., et al. (2023). Efficient Interfacial Electron Transfer Induced by Hollow-structured $ZnIn_2S_4$ for Extending Hot Electron Lifetimes. Energy Environ. Sci. *16*, 3462-3473. 10.1039/D3EE01522J.
